# Interpretation of allele-specific chromatin accessibility using cell state–aware deep learning

**DOI:** 10.1101/gr.260851.120

**Published:** 2021-06

**Authors:** Zeynep Kalender Atak, Ibrahim Ihsan Taskiran, Jonas Demeulemeester, Christopher Flerin, David Mauduit, Liesbeth Minnoye, Gert Hulselmans, Valerie Christiaens, Ghanem-Elias Ghanem, Jasper Wouters, Stein Aerts

**Affiliations:** 1 VIB-KU Leuven Center for Brain and Disease Research, 3000 Leuven, Belgium;; 2 KU Leuven, Department of Human Genetics KU Leuven, 3000 Leuven, Belgium;; 3 Cancer Genomics Laboratory, The Francis Crick Institute, London NW1 1AT, United Kingdom;; 4 Institut Jules Bordet, Université Libre de Bruxelles, 1000 Brussels, Belgium

## Abstract

Genomic sequence variation within enhancers and promoters can have a significant impact on the cellular state and phenotype. However, sifting through the millions of candidate variants in a personal genome or a cancer genome, to identify those that impact *cis*-regulatory function, remains a major challenge. Interpretation of noncoding genome variation benefits from explainable artificial intelligence to predict and interpret the impact of a mutation on gene regulation. Here we generate phased whole genomes with matched chromatin accessibility, histone modifications, and gene expression for 10 melanoma cell lines. We find that training a specialized deep learning model, called DeepMEL2, on melanoma chromatin accessibility data can capture the various regulatory programs of the melanocytic and mesenchymal-like melanoma cell states. This model outperforms motif-based variant scoring, as well as more generic deep learning models. We detect hundreds to thousands of allele-specific chromatin accessibility variants (ASCAVs) in each melanoma genome, of which 15%–20% can be explained by gains or losses of transcription factor binding sites. A considerable fraction of ASCAVs are caused by changes in AP-1 binding, as confirmed by matched ChIP-seq data to identify allele-specific binding of JUN and FOSL1. Finally, by augmenting the DeepMEL2 model with ChIP-seq data for GABPA, the TERT promoter mutation, as well as additional ETS motif gains, can be identified with high confidence. In conclusion, we present a new integrative genomics approach and a deep learning model to identify and interpret functional enhancer mutations with allelic imbalance of chromatin accessibility and gene expression.

Understanding the functional consequences of noncoding variants is still a fundamental challenge in human genetics. Genome-wide association studies indicate that almost 90% of disease-related variants reside in noncoding regions (Hindorff et al. 2009; Maurano et al. 2012), and these regions are enriched for transcription factor (TF) binding sites (Khurana et al. 2013). A large body of work has been devoted to identifying noncoding variants that alter gene regulation by linking them to functional genomics data (Gaffney et al. 2012; The GTEx Consortium 2015; Chen et al. 2016; Banovich et al. 2018). Broadly, the approaches taken to address this problem can be classified into two groups. The first one is quantitative trait loci (QTL) analysis, in which a variant is correlated to a cellular trait (e.g., expression, binding, accessibility) across a large number of samples. This strategy is widely used with expression, chromatin immunoprecipitation (ChIP), and chromatin accessibility data for detecting, respectively, QTL associated with gene expression (eQTL), TF binding (bQTL) (Kilpinen et al. 2013), histone modifications (hQTL) (McVicker et al. 2013), or chromatin accessibility (caQTL) (Maurano et al. 2015). This type of analysis is cost-efficient and can be conducted with array-based data but requires large sample sizes, because effect sizes are usually low (Do et al. 2017). Moreover, the resolution is typically too low to pinpoint a single variant owing to linkage disequilibrium (typically spanning 10 to 100 kb) (Do et al. 2017). Additionally, structural variation and rare variants (minor allele frequency < 0.05) are often ignored in these studies (Chen et al. 2016; Audano et al. 2019). The alternative approach is to assess allelic imbalance at a heterozygous site directly. This allele-counting approach has been extensively used with RNA-seq data to identify allele-specific expression (Castel et al. 2015) but is also applicable to other types of functional genomics data (Rozowsky et al. 2011; Chen et al. 2016). Here, the strategy relies on finding the allelic origin of the observed signal. Unlike QTL analysis, this approach does not depend on large sample sizes and can be used to find rare or even de novo regulatory variants; however, it requires higher genomic coverage and more complex data processing. Technical issues inherent to alignment and variant calling procedures such as reference bias (i.e., reads originating from the reference allele map better than those containing the variant), ambiguous alignments, and copy number alterations need to be addressed in order to obtain accurate measures of allelic imbalance (Rozowsky et al. 2011; Chen et al. 2016; de Santiago et al. 2017). The use of personalized diploid genomes instead of a haploid reference has been suggested to prevent some of these technical biases (Rozowsky et al. 2011; Castel et al. 2015; Chen et al. 2016).

Both QTL analysis and inference of allelic imbalance can lead to the identification of candidate gene regulatory variants. However, they typically yield little information as to the precise regulatory mechanisms affected by these variants. More than 70% of noncoding variants associated with common diseases overlap with TF binding sites (Maurano et al. 2012); however, studies so far showed that the majority of the variants associated with allele-specific enhancer activity cannot be explained by TF motif alterations (Degner et al. 2012; Kilpinen et al. 2013; Maurano et al. 2015; Waszak et al. 2015; Deplancke et al. 2016; Kumasaka et al. 2016; Tehranchi et al. 2019). This might be because of the inadequacy of current TF motif models (such as position weight matrices [PWMs]) that do not take other enhancer features into account, such as flanking sequence context, DNA shape, or combinatorial TF binding (Inukai et al. 2017). By leveraging the extensive chromatin and TF binding data available, machine-learning approaches hold promise to predict TF-bound regions and chromatin changes with single-nucleotide resolution. However, these models require correct training and rigorous validation and are typically trained either for a single TF (Alipanahi et al. 2015; Lee 2016; Quang and Xie 2019; Avsec et al. 2021) or for hundreds of epigenomic features (Zhou and Troyanskaya 2015; Kelley et al. 2016). These models tend to be cell type–specific, resulting in reduced performance when applied to other cell types (Banovich et al. 2018). We have previously shown that specialized deep learning models can outperform generic ones at predicting regulatory features and the effect of sequence variation across species (Minnoye et al. 2020).

Here, we perform a comprehensive analysis of 10 melanoma whole genomes to identify and characterize functional noncoding variants. By integrating sample-matched phased whole-genome sequencing (WGS), assay for transposase-accessible chromatin using sequencing (ATAC-seq), ChIP against H3K27ac (ChIP-seq), and transcriptome sequencing (RNA-seq) data, we identify allele-specific regulatory changes. To interpret how sequence variation affects the gain or loss of TF binding sites, we used a deep learning model, called DeepMEL2, that is trained on different melanoma cell states. We investigate the benefits and limitations of cell state–specific deep learning and motif analysis to unravel how enhancer mutations affect gene regulation.

## Results

### Identification of ASCAVs using linked-read genome sequencing and ATAC-seq

**Figure 1.**
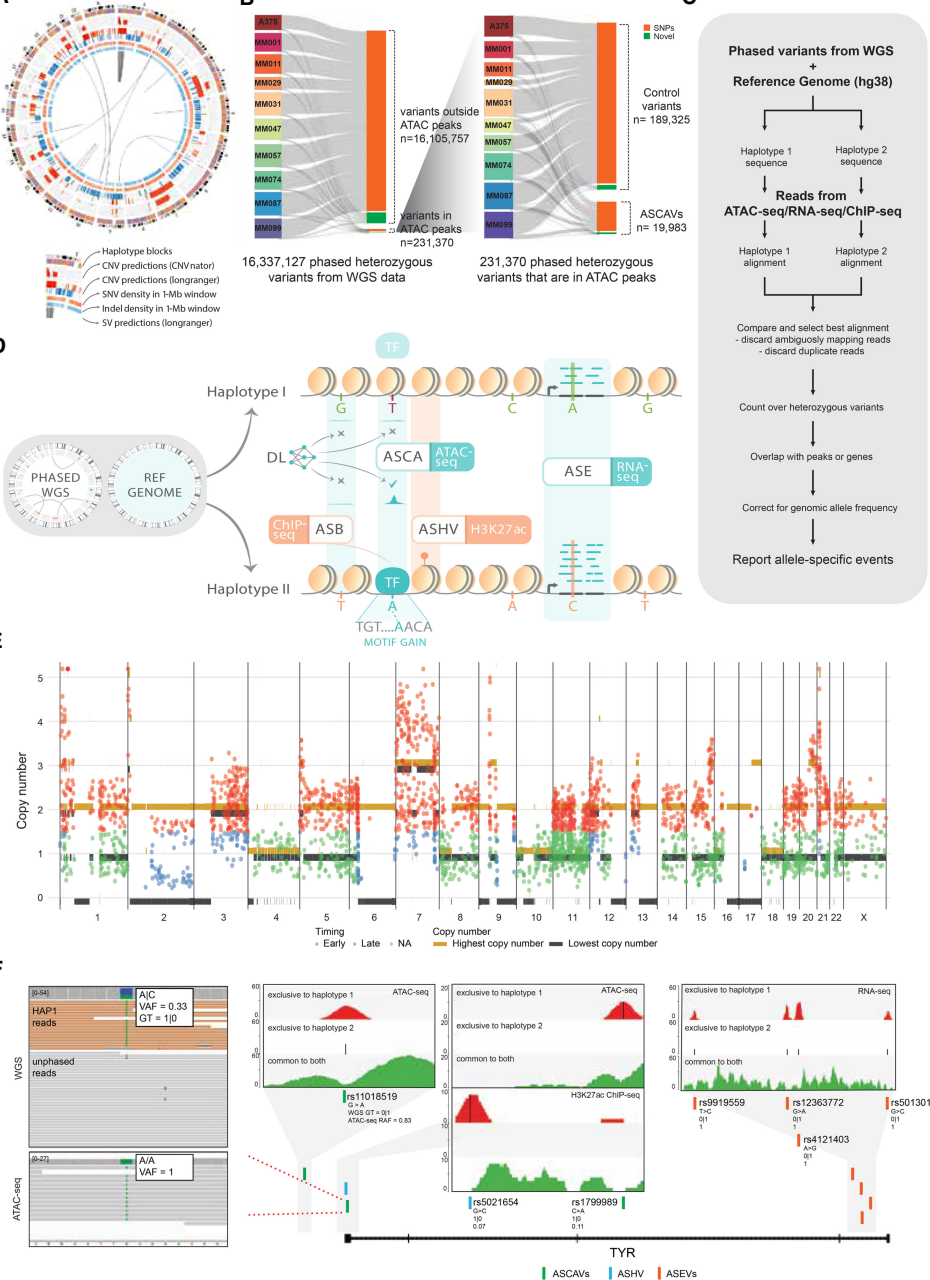
Detection of allele-specific chromatin accessibility. (*A*) Circos plot for sample MM074. Circos plots for the remaining samples are shown in Supplemental Figure S1. (*B*) Sankey diagram of the number of variants that went through our ASCAV discovery pipeline. (*C*) Analysis pipeline for identification of allele-specific events from matched phased whole-genome data and functional genomics data (ATAC-seq, RNA-seq, or ChIP-seq). (*D*) Phased whole-genome sequencing (WGS) is applied to 10 melanoma cell lines and is used together with the reference genome to create personalized diploid genomes. Matched ATAC-seq, RNA-seq, and ChIP-seq data (against H3K27ac mark and transcription factors [TFs]) are used to detect allelic imbalance in chromatin accessibility (ASCA), gene expression (ASE), histone acetylation (ASHV), or allele-specific binding (ASB). By combining a melanoma-specific deep learning model (DeepMEL2) and motif discovery, *cis*-regulatory variants are predicted. (*E*) Genome-wide allele-specific copy number is shown for sample MM074. Superposed are the identified ASCAVs in this cell line, of which the mutation copy number is plotted. The color of the ASCAVs indicates whether they can be classified as either early or late. If their copy number context does not allow timing, they are labeled “na.” Allele-specific copy numbers for the remaining samples are shown in Supplemental Figure S4. (*F*) Concordant allele-specific events are detected around *TYR*, a gene encoding an enzyme involved in pigmentation. *Inset* shows the reads from whole-genome and ATAC-seq data for one of the allele-specific SNPs (rs1799989). Whole-genome data indicate a haplotype 1–specific heterozygous SNP (i.e., GT = 1|0) with a variant allele frequency of 0.33, whereas ATAC-seq data indicate the reads are coming from one allele (haplotype 1). There are a further six allele-specific variants in *TYR* that are either haplotype 1 (i.e., GT = 1|0) or haplotype 2 (i.e., GT = 0|1) specific in the WGS data, yet all the variants manifest a haplotype-specific activity in matched functional genomics data. The *inset* plots for all these seven variants show ATAC-seq, H3K27ac ChIP-seq, or RNA-seq reads in these loci segregated into haplotypes. Reads mapping exclusively to haplotype 1 are shown at the *top* (red), whereas the ones mapping exclusively to haplotype 2 are shown in the *middle* (blue). We can detect exclusive mapping only at the variant locations; hence, the majority of the reads map equally well to both haplotypes and are shown at the *bottom* (green). Additionally, reference allele fractions (RAFs) are shown for all the variants (corrected RAFs are obtained via BaalChIP for ASCAVs and ASHV).

We obtained haplotype-resolved WGS data of 10 patient-derived melanoma cultures (MM lines) using linked-read technology from 10x Genomics (Fig. 1A; Supplemental Fig. S1; Supplemental Tables S1, S2). Samples were sequenced to an average depth of 38×, apart from MM087 and MM099, which were sequenced more deeply (68× and 133× coverage, respectively). We also profiled the chromatin accessibility of the same melanoma lines using Omni-ATAC-seq (nine samples were reanalyzed from Wouters et al. 2020, whereas data for A375 were generated in this study).

We find a total of 16 million phased variants across the 10 genomes (Fig. 1B) and pinpoint as likely somatic in origin between 206,724 and 304,754 of these per sample, based on their absence from the Genome Aggregation Database (gnomAD; v3.0). To dissect the contributions of distinct mutational processes, we estimated exposures to the COSMIC v3 single-base substitution signatures. We used the Bayesian approach implemented in SigFit (Gori and Baez-Ortega 2020) and fitted those signatures that have previously been reported as active in melanoma (Supplemental Fig. S2). Overall, although a mean 10.9% of somatic mutations showed the unique footprint of UV damage (COSMIC v3 SBS7a–d and -38; range 10.8%–26.2%), 88.5% found its origin in the endogenous “clock-like” mutational processes SBS1, -5, and -40 (range 73.7–98.7%) (Supplemental Fig. S2; Alexandrov et al. 2020). Note that in addition to true somatic SNVs having arisen during tumorigenesis, these “clock-like” variants will contain contributions from germline SNPs that were absent from gnomAD, as well as SNVs having arisen during passaging in culture. Only an average of 0.5% of somatic mutations were assigned to the remaining signatures (range 0.1%–2.3%).

Next, we constructed a personal genome for each sample to optimize the mapping accuracy at variant positions (see Methods). By combining these personal genomes with ATAC-seq, we found 231,370 variants (of the 16 million) that overlap with ATAC-seq peaks. We then tested each of these variants for allelic imbalance of the overlapping ATAC-seq signal using a modified *alleleseq* pipeline, yielding allele-specific chromatin accessibility variants (ASCAVs; Methods) (Fig. 1C,D; Chen et al. 2016). Although binomial or beta-binomial tests are used for the detection of allele-specific events, the ubiquitous presence of copy number aberrations in cancer genomes violates the assumptions of these methods. Therefore, we plugged in the Bayesian framework of BaalChIP, which specifically addresses this problem (de Santiago et al. 2017). BaalChIP enabled us to correct the allelic ratios observed in ATAC-seq reads using the genomic allelic ratios from the WGS data to correct for the extensive copy number variation and frequent whole-genome doubling in these lines (Supplemental Figs. S3, S4). This pipeline resulted in 19,983 significant ASCAVs (8.6% of the variants that overlap with an ATAC-seq peak) across the 10 genomes (range 451–7183 per sample) (Supplemental Tables S3, S4). The majority of ASCAVs are unique to one MM line, and a small proportion is shared between multiple samples (1073 out of 19,983; 5.4%) (Supplemental Fig. S5). Only two of the shared ASCAVs are called discordantly between the samples, all of which are known multiallelic polymorphic SNPs (rs138784536, rs9880846), illustrating the accuracy of the ASCAV pipeline. We also assembled a set of control heterozygous variants within the ATAC-seq peaks that show no allelic bias. The genomic distribution of both sets, ASCAVs and control variants, is highly similar (Supplemental Fig. S6; Supplemental Table S5).

Even though most ASCAVs are germline polymorphisms (88.9%), 2201 ASCAVs are likely somatic (i.e., absent from gnomAD v3.0). Somatic variants hence appear more likely to constitute ASCAVs than do germline variants (chi-square test per sample, all *P* ≤ 3.28 × 10^−8^). This may be explained in part by increased local mutation rates at TF-bound motifs, negative selection in the germline, and/or positive selection in the tumor. Furthermore, if a mutational process would be more (or less) prone to introduce ASCAVs, this may be detectable as a disproportionate contribution of the corresponding mutation signature to somatic ASCAVs compared with all somatic variants. We therefore re-estimated the mutational signature exposures specifically from the somatic ASCAVs and contrasted these estimates with the signature exposures obtained from all somatic variants. No consistent differences in signature activities could be detected (Supplemental Fig. S7). Nevertheless, the five samples with the highest overall proportion of SBS7a mutations showed a smaller contribution of this type of mutations to ASCAVs, suggesting UV-induced lesions do not systematically affect chromatin accessibility.

To further evaluate whether our ASCAV detection pipeline is robust to copy number variation, we inferred allele-specific copy number from the WGS data (Van Loo et al. 2010), evidencing extensive aneuploidy (Fig. 1E; Supplemental Fig. S4). In addition, using sample ploidy and the fraction of the genome with loss of heterozygosity, we were able to classify eight of our 10 lines as having undergone a whole-genome doubling (Supplemental Fig. S8; Dentro et al. 2021). By considering the number of chromosome copies carrying an ASCAV, we can time the variants with respect to copy number gains: If a variant arose on a chromosome before its duplication, it will be duplicated as well (mutation copy number ≥ 2, an “early” variant). If it arose after, only one copy will be present (mutation copy number = 1, a “late” variant). In regions with loss of heterozygosity or gains on both alleles, these two scenarios can be readily distinguished (Gerstung et al. 2020). Apart from a reduction in regions with loss of heterozygosity (i.e., only somatic and no germline variants can be tested for allelic skewing), ASCAVs are called across all copy number states, and both early and late variants are detectable as ASCAVs, confirming that our pipelines are robust in the face of copy number changes (Fig. 1E).

### A subset of ASCAVs overlaps with allele-specific gene expression and allele-specific histone modifications

To investigate whether allele-specific chromatin accessibility is associated with allelically skewed gene expression or histone modifications, we analyzed matching RNA-seq and H3K27ac ChIP-seq data for all 10 samples (Verfaillie et al. 2015). RNA-seq and ChIP-seq reads were processed with the same analysis pipeline (Fig. 1C). For identifying allele-specific expression variants (ASEVs) in the presence of copy number alterations, we used a beta-binomial test of the RNA-seq allele counts, in which the shape of the beta distribution is informed by the corresponding WGS allele counts (Methods). We identified 11,578 distinct autosomal ASEVs, associated with 6029 genes (Supplemental Table S6). One gene, *MAP2K3* (also known as MEK3), shows ASE in all 10 samples and was previously reported to be allele specifically expressed in various human and mouse tissues (Tuskan et al. 2008; Kukurba et al. 2014). Globally, ASCAVs are enriched near genes with ASE (*P*-value = 0.005; Fisher's exact test) with 6% of ASCAVs located in promoters (<2 kb upstream of a TSS) or introns of ASE genes (Supplemental Table S7).

We also tested for allele-specific H3K27ac ChIP-seq signal using the same pipeline coupled to BaalChIP. Across the 10 lines, we identified 4016 allele-specific histone variants (ASHVs) (Supplemental Table S8), 343 are both ASCAVs and ASHVs, and an additional 170 are within 1 kb of an ASCAV. Similar to ASEVs, ASCAVs are enriched near ASHVs compared with control variants (*P*-value < 2.2 × 10^−16^; Fisher's exact test) (Supplemental Table S9). When combined, there are 1589 ASCAVs that are either close to a gene with ASE or close to an ASHV, and 89 of them show significant changes on all three levels (odds ratio of 1.6 and 3.9, respectively, compared with control variants, with *P*-value < 2.2 × 10^−16^). One such example is observed near the pigment-associated factor *TYR* (Fig. 1F), in which four loci show allele-specific events on all three levels (ATAC-seq, H3K27ac ChIP-seq, and RNA-seq). Taken together, our finding that a significant fraction of ASCAVs is linked with ASHVs and ASE supports their functional relevance.

### TF motifs are enriched on ASCAVs, with AP-1 being dominant

Next, we investigated whether ASCAVs affect TF binding sites. We evaluated a variety of regulatory sequence analysis tools to assess which ASCAVs may have arisen through direct *cis*-regulatory changes, such as gains or a losses of TF binding sites (Fu et al. 2014; Maurano et al. 2015; Deplancke et al. 2016), and which ASCAVs are more likely to result from indirect events. We first evaluate simple models, namely, PWMs before moving on to more advanced deep learning–based models and comparing their prediction accuracy.

**Figure 2.**
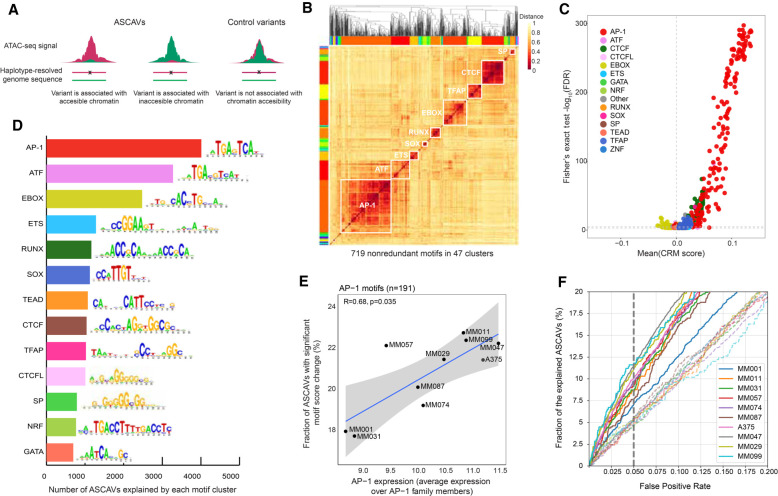
TF motif enrichment on ASCAVs. (*A*) Selection of ASCAVs and control variants used to assess the association between sequence content and allele-specific accessibility. (*B*) Heatmap showing the clustering of all 719 ASCAV-enriched motifs into 47 families (color-coded margins). The 13 major families are labeled with their cognate TF on the diagonal. (*C*) Scatter plot of motifs that are associated with chromatin accessibility. Each dot indicates a motif and is colored based on the motif cluster to which they belong. The *x*- and *y*-axes represent the delta cluster-buster motif score and the negative log-scaled FDR corrected *P*-value, respectively. (*D*) Bar plot showing the number of ASCAVs explained by each motif cluster. For each family, the consensus motif is shown. (*E*) Scatter plot of the average expression of AP-1 family members (JUN, JUNB, JUND, FOS, FOSB, FOSL1, FOSL2) and the fraction of ASCAVs that affects an AP-1 binding site. Correlation coefficient (Kendall's tau) and *P*-value are shown. (*F*) Fractions of ASCAVs explained at different false-positive rates are shown as curves for each MM line. Dashed lines represent the control for each MM line, where labels of ASCAVs and control variants are shuffled.

Binding site predictions using PWMs are notorious for their high false-positive rates (Wasserman and Sandelin 2004), and this problem is aggravated as our collection of PWMs is very large (more than 22,000 PWMs) (Janky et al. 2014). To overcome this problem, we asked whether, for any given TF, multiple binding sites are gained or lost in a sample or across the cohort. This provides a statistical cue, as we can exploit motif enrichment across all variants, testing which PWM yields a disproportionate number high “delta-PWM scores,” compared with the control variants. A similar motif enrichment technique has been applied before to identify pioneer factors from chromatin accessibility QTL data (Jacobs et al. 2018). Out of all 22,000 motifs tested, 719 are significantly altered by ASCAVs compared with control SNPs (Fisher's exact test, FDR 0.05) (Fig. 2A). As our collection of motifs is highly redundant (multiple PWMs are present per TF), we clustered the 719 significant PWMs into 47 distinct families. We then focused on 13 of these clusters for which the associated TF is known and that contained at least six motifs (Fig. 2B). This analysis revealed the AP-1 family as the top hit, with a total of 191 enriched PWMs (FET-adjusted *P*-value threshold 0.05) in 4011 allele-specific ATAC-seq peaks across the 10 samples (Fig. 2C,D; Supplemental Table S10; Supplemental Fig. S9). We observed a significant correlation (Kendall's tau 0.68 with *P*-value = 0.035) between expression of AP-1 factors (all JUN and FOS paralogs together) and the fraction of explainable ASCAVs per sample (Fig. 2E). Indeed, MM lines of the mesenchymal subtype (MES; MM099, MM047, and MM029) have higher AP-1 activity and more AP-1 motif gains and losses at ASCAVs compared with MM lines of the melanocytic subtype (MEL; MM031 and MM001). MM011 represents an exception in this case, being of the MEL subtype but with high AP-1 activity. The remaining lines (MM087, MM057, and MM074) are in an intermediate state (Wouters et al. 2020). Overall, these findings suggest that AP-1 binding sites are strongly correlated with changes in chromatin accessibility, and confirm the power of allele-specific chromatin accessibility profiling to identify both gain- and loss-of-function enhancer mutations. AP-1 has, indeed, been reported to act as a pioneer factor, resulting in nucleosome displacement at enhancers in murine mammary epithelial cells (Biddie et al. 2011).

By using the entire set of 719 enriched motifs, we calculated the delta-PWM score across all ASCAVs. This allows us to evaluate the sensitivity and specificity of the PWM approach for predicting which variants induce allele-specific chromatin accessibility (Fig. 2F). At 95% specificity, 1919 variants are predicted to be ASCAVs. Finally, we also tested whether some motif gains or losses can be negatively associated with accessibility, that is, the delta-PWM and accessibility are negatively correlated. We only identified motifs linked to TFs of the ZEB/SNAI family, which are known repressors in the neural crest lineage, including in melanomas (Supplemental Fig. S10) (Postigo and Dean 1999; Postigo et al. 1999; Peinado et al. 2007; Caramel et al. 2013; Denecker et al. 2014).

In conclusion, motifs of the relevant TFs are enriched at ASCAVs, suggesting that ∼9.6% of variants in ATAC-seq peaks create or break a binding site. In turn, such motif gains or losses likely underlie the observed allele-specific chromatin accessibility signals.

### A cell state–aware deep learning model can interpret ASCAVs

We next tried to improve the accuracy obtained with the PWM approach using more advanced enhancer modeling. Machine-learning models can be trained on enhancers and take flanking sequence information into account. Examples of deep learning models are Basset (Kelley et al. 2016) and DeepSEA (Zhou and Troyanskaya 2015), which are available in Kipoi (Avsec et al. 2019) and can readily be applied to score *cis*-regulatory variants. These generic models have been trained on large collections of epigenomic data (DeepSEA was trained on 919 cell type–specific epigenomic features; Basset was trained on DNase-seq from 164 cell types), allowing their application to “any” cell type. Their prediction accuracy on our MM lines (to discriminate ASCAVs from control variants) is usually higher than that of the motif-based approach (Supplemental Fig. S11). Particularly on the MES lines, Basset and DeepSEA achieve high accuracy, explaining 14%–16% of ASCAVs by motif changes, at 95% specificity. This is likely because the training data were rich in AP-1-bound enhancers, which are well represented in the ENCODE repositories on which DeepSEA and Basset were trained.

Next, we train our own deep learning model that takes the main melanoma cell states into account, namely, the melanocytic state (MEL) expressing melanocyte-specific TFs and pigmentation genes, and the mesenchymal-like state (MES) in which cells are more invasive and therapy resistant (Verfaillie et al. 2015; González-Blas et al. 2019; Wouters et al. 2020). In our previous work (Minnoye et al. 2020), we trained a deep learning model, DeepMEL, on ATAC-seq from a cohort of 16 melanoma samples, including the 10 MM lines used in this work. Applying DeepMEL to discriminate ASCAVs from control variants outperforms the generic models (Basset and DeepSEA) for the MEL but not MES lines. This is likely because the generic models were trained on a larger data set with a high amount of MES-like genomic enhancers. In contrast, melanocyte and MEL-melanoma states are likely underrepresented in ENCODE, resulting in models that are not fully “aware” of this regulatory program.

**Figure 3.**
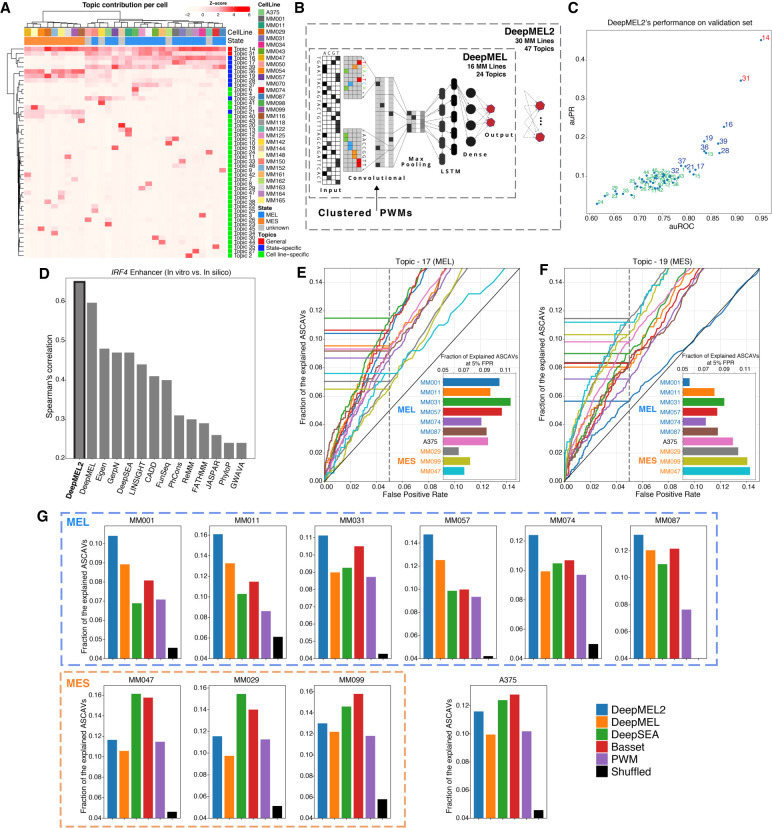
Cell state–aware DeepMEL2 can interpret ASCAVs. (*A*) Normalized cisTopic cell-topic heatmap of 30 melanoma cell lines showing general, state-specific, and cell line–specific sets of coaccessible regions. (*B*) Schematic overview of DeepMEL2 highlighting improvements compared with DeepMEL. (*C*) Scatter plot of auROC and auPR values shows the performance of DeepMEL2 on each topic. Promoter, state-specific, and cell line–specific topics are represented by red, blue, and green colors, respectively. (*D*) Performance of DeepMEL2 and other models at predicting variant effects on *IRF4* enhancer activity. (*E*,*F*) Curves indicate fractions of ASCAVs explained by Topic-17 score (MEL; *E*) and Topic-19 score (MES; *F*) at different false-positive rates for each MM line. Bar chart *insets* show the exact fraction of the explained ASCAVs at 5% false-positive rate. (*G*) Bar charts showing the fraction of ASCAVs explained at 5% false-positive rate for each MM line using either DeepMEL2, DeepMEL, DeepSEA, Basset, and PWM. The black bar represents the fraction when ASCAVs and control variants are shuffled.

We then asked whether we could further improve DeepMEL, including additional ATAC-seq data (and further below also ChIP-seq). We extended our DeepMEL cohort with 14 new samples to a total of 30 melanoma lines. A cisTopic (González-Blas et al. 2019) analysis on this larger cohort identifies 47 *cis*-regulatory topics, in which two of them are generally accessible across all cell lines (Topic-14 and Topic-31) and nine state-specific MEL and MES topics (Fig. 3A). We also enhanced the deep learning framework by including the 283 known clustered and partitioned PWMs from the JASPAR database (Fornes et al. 2020) in the convolutional filters that serve as priors (Fig. 3B). After training DeepMEL2 on the 47 topics, we evaluated its classification performance on left-out data (Supplemental Fig. S12). Particularly promoter topics, MEL-topics, and MES-topics achieve high performance, whereas cell line–specific topics are difficult to predict (Fig. 3C). For the latter, we believe this is because the cell line–specific topics represent sample-specific copy number variation rather than differentially accessible regions (Supplemental Fig. S4). Next, we applied in silico saturation mutagenesis on the MEL-type *IRF4* enhancer. This explainable AI technique, in which each possible mutation in the enhancer sequence is evaluated by reclassification using the model, highlights the outperformance of DeepMEL2 compared with DeepMEL (Fig. 3D).

Next, we used DeepMEL2 to score all ASCAVs (Methods). On the six MEL lines, DeepMEL2 identifies more ASCAVs compared with all the other methods at the same false-positive rate (Methods) (Fig. 3G; Supplemental Fig. S11). When we score ASCAVs by using a MEL-specific topic (Topic-17), which represents MEL enhancers, explainable mutations occur more frequently in the samples of the melanocytic subtype, in which MEL enhancers are operational (Fig. 3E). A MES-specific topic (Topic-19), on the other hand, is mostly affected in samples of the MES (Fig. 3F). Note that, in agreement with the motif enrichment analysis, AP-1 motif changes (the main drivers of the MES scores) (Fig. 2C,D) are also found enriched for ASCAVs in the melanocytic lines, except for MM001, which has no AP-1 activity (Supplemental Fig. S9).

In conclusion, additional enhancement and training of DeepMEL2 further improves prediction of functional *cis*-regulatory changes, particularly on melanoma samples of the MEL subtype, and provides high-resolution insight into precise enhancer changes.

### DeepMEL2 predictions on ASCAVs are confirmed by allele-specific TF binding

**Figure 4.**
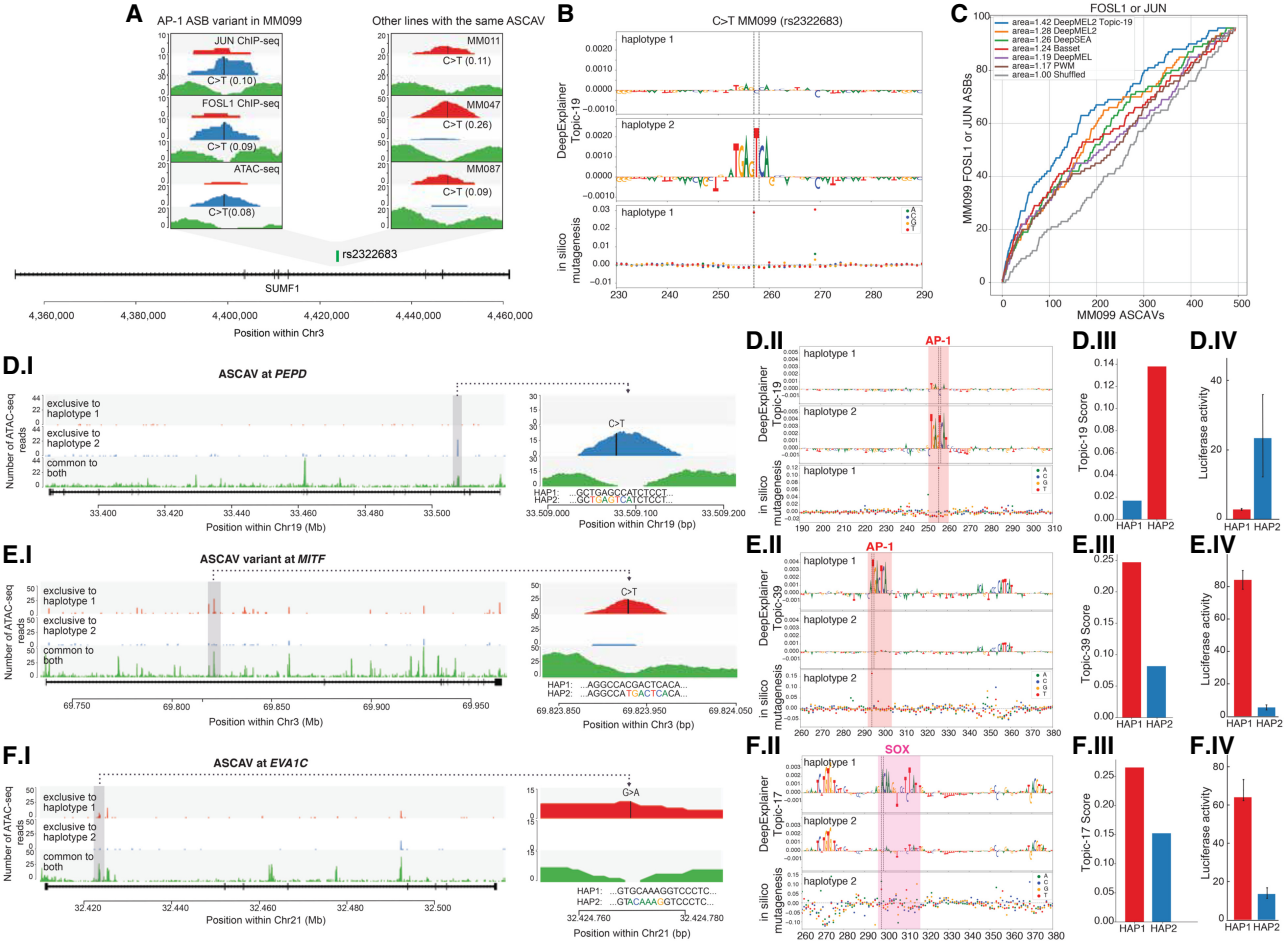
Model explanation and experimental validation of three *cis*-regulatory variants. (*A*) C > T intronic SNP (rs2322683) in *SUMF1* is an ASCAV and AP-1 ASB (JUN and FOSL1 ChIP-seq data sets). (*Left*) Haplotypes 1 and 2 and unphased reads (color-coded) from this locus in MM099 JUN and FOSL1 ChIP-seq and ATAC-seq reads. (*Right*) Same locus in three additional MM lines (MM011, MM047, and MM087) in which rs2322683 is also inferred as an ASCAV. WGS genotypes (GT) and BaalChIP allele ratios are shown in parentheses. (*B*) DeepExplainer plot of the rs2322683 locus (position indicated with dashed lines), where the height of the nucleotides indicates their importance for the final prediction. Scoring using Topic-19 on both haplotypes shows C > T substitution generates an AP-1 binding site. In silico saturation mutagenesis on the reference sequence reveals the effect of each possible variant as a delta Topic-19 prediction score. (*C*) The curves represent the number of FOSL1 or JUN ASB variants found among the *top*-*n* MM099 ASCAVs ranked by the maximum delta prediction score of the different models. (*D–F*) Each row showcases the following: (*I*) an ASCAV and its allele-specific accessibility peak, (*II*) DeepExplainer and in silico mutagenesis results of the two haplotypes, (*III*) the DeepMEL2 score for both haplotypes, and (*IV*) the luciferase enhancer-reporter activity for both haplotypes. (*D*) C > T intronic variant in *PEPD* is identified as an ASCAV and predicted to generate an AP-1 binding site, with an increase in MES enhancer score. The in silico mutagenesis plot shows that only a single mutation to T at position 269 increases the MES enhancer prediction significantly, and this is exactly the location of the ASCAV. (*E*) C > T intronic variant in *MITF* is identified as an ASCAV and predicted to generate an AP-1 binding site. (*F*) G > A intronic variant in *EVA1C* is identified as an ASCAV and predicted to generate a SOX10 binding site.

We predicted a large fraction of allele-specific AP-1 binding sites that are associated with an allele-specific ATAC-seq peak. To test whether AP-1 factors indeed bind preferentially to the predicted allele, we performed ChIP-seq against four AP-1 family members (JUN, JUNB, FOS, FOSL1) that are expressed in the MES-type MM099 line. The ChIP-seq peaks of all four data sets are enriched for the AP-1 motif (Supplemental Fig. S13). The FOSL1 and JUN ChIP-seq yield the highest quality peaks, suggesting that these play a role in MM099 and that these antibodies are of high quality (Supplemental Fig. S13). By using the pipeline developed above to infer ASCAVs, we now identify 583 significant allele-specific binding (ASB) events for JUN and 241 for FOSL1 (JUNB and FOS yield only 138 ASBs in total), and some of them are identified as ASCAVs in other cell lines as well (Fig. 4A). The MES-specific topics are able to predict allele-specific AP-1 binding events (Fig. 4B). When we rank all MM099 ASCAVs by their maximum score from the different models (i.e., delta between the two alleles for the PWM approach, Basset, DeepSEA, DeepMEL, and DeepMEL2), we find that DeepMEL2 performs best at enriching for ASB events (Fig. 4C). This means that a significant fraction of the ASCAVs with high DeepMEL2 delta scores are indeed ASB for JUN or FOSL1. Note that whereas Basset and DeepSEA are better at distinguishing ASCAVs from control variants in MEL099, this is not the case for predicting ASB. Because DeepMEL2 was trained to distinguish 47 different melanoma *cis*-regulatory topics, we can also score ASCAVs using specific topics. Leveraging the best performing MES topic (Topic-19) indeed further improves the prediction of allele-specific AP-1 binding (Fig. 4C). Note that we did not search specifically for AP-1 sites but rather exploit the regulatory topic of the matching cell state to score the genomic variants.

In a second validation experiment, we evaluated the putative effect of ASCAVs on enhancer activity. Our phased genomes allow direct linking of ASCAVs to allele-specific expression of nearby genes. A total of 6.5% of all ASCAVs that can be explained by DeepMEL2 are located in the promoter or body of genes with ASE. Therefore, these enhancer mutations may underlie the expression imbalance of the target gene. To further examine this, we selected three enhancers in MM057 for which the predicted target gene shows ASE. The first two examples, *PEPD* and *MITF*, have a DeepMEL2-predicted AP-1 motif gain (Fig. 4D,E). Luciferase reporter assays in this cell line, using sequences of both haplotypes, confirm the potency of these variants to drive enhancer activity and gene expression only when the AP-1 site is present (Fig. 4D,E). The third example is an enhancer in the first intron of *EVA1C* with a predicted SOX10 motif gain. This variant (rs2833812) is identified as a phased heterozygous SNP in four lines (MM031, MM057, MM074, MM087) and results in allele-specific accessibility in all cases (Supplemental Fig. S14). Again, when assessed in a luciferase reporter assay (in MM057), only the enhancer sequence that carries the allele generating the SOX10 motif is able to drive luciferase expression (Fig. 4F; Supplemental Fig. S17). This confirms that enhancer mutations associated with changes in chromatin accessibility can have an effect on the expression of nearby genes.

### Analysis of *TERT* promoter mutations by augmenting DeepMEL2 with ChIP-seq data

**Figure 5.**
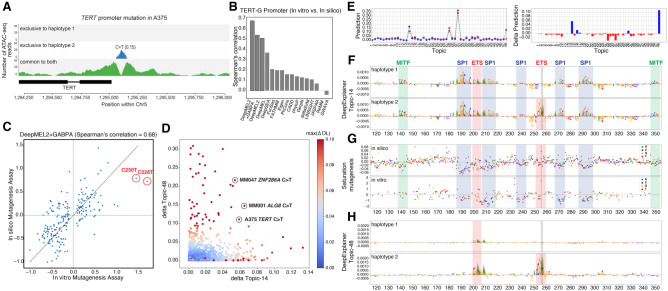
Analysis of *TERT* promoter mutations. (*A*) *TERT* promoter hotspot mutation in A375 is detected as an ASCAV as evidenced by ATAC-seq reads segregated into haplotypes (color-coded). In A375, haplotype 2 harbors the mutant allele T (according to WGS data) (see Supplemental Fig. S16), and ATAC-seq evidences exclusive accessibility for this allele. The corrected reference ATAC-seq allele ratio is indicated in parentheses. (*B*) Bar chart of model variant effect prediction performance on *TERT* promoter activity assessed by experimental saturation mutagenesis. (*C*) Scatter plot showing the effect of each variant in the in vitro (*x*-axis) and in silico (*y*-axis) mutagenesis of the *TERT* promoter. The two hotspot gain-of-function mutations are highlighted. (*D*) Scatter plot of delta Topic-14 score (promoter topic) versus delta Topic-48 score (GABPA topic) of all ASCAVs from 10 MM lines calculated by using the DeepMEL2 + GABPA model. ASCAVs are colored by their maximum delta prediction score. The *TERT* mutation of A375, as well as two newly predicted GABPA gains in MM047 and MM001 that are discussed in the text, are encircled. (*E*) The DeepMEL2 prediction score for each topic for both the haplotype 1 (red) and haplotype 2 (blue) of the A375 *TERT* locus is shown on the *left*, and the delta prediction scores between two haplotypes are shown on the *right*. The delta prediction scores for both Topic-14 (promoter topic) and Topic-48 (GABPA topic) are above the 0.05 detection threshold. (*F*,*H*) Haplotype-specific DeepExplainer plots of the A375 TERT promoter locus by using Topic-14 (*F*) and Topic-48 (*H*), annotated with the corresponding TFs. (*G*) Comparison of in silico (*top*; DeepMEL2 delta Topic-14 prediction scores) and in vitro (*bottom*; fold change in promoter activity) saturation mutagenesis assay. Each variant is color-coded.

As a final analysis, we asked whether DeepMEL2 can identify oncogenic mutations in the *TERT* promoter (Horn et al. 2013; Huang et al. 2013). Two *TERT* promoter hotspots are recurrently mutated (C228T and C250T) across a large fraction of cancers of the central nervous system (43%), bladder cancer (59%), melanoma (29%), and other cancer types (Vinagre et al. 2013). These gain-of-function mutations create a binding site for the ETS-family TFs, notably GAPBA, leading to up-regulation of the *TERT* oncogene (Bell et al. 2015). The A375 cell line contains one of these mutations, which is predicted as an ASCAV (Fig. 5A).

First, we evaluated the accuracy of DeepMEL2 and other methods to predict functional changes across the *TERT* promoter, by comparing the predictions to a previously published saturation mutagenesis screen performed in a glioblastoma cell line (Kircher et al. 2019). In silico saturation mutagenesis of the *TERT* promoter, scored with DeepMEL2, correlates strongly (54%) with the experimental data, outperforming other methods (Fig. 5B). Despite the ability of DeepMEL2 to interpret overall *cis*-regulatory variation in the *TERT* promoter, the model does not predict the oncogenic gains of GABPA sites themselves to cause an increase in enhancer activity.

In an attempt to improve interpretation of oncogenic *TERT* mutations, we retrained DeepMEL2 by adding a 48th topic representing GABPA binding. Particularly, we labeled all ATAC-seq peaks that overlap with GABPA ChIP-seq peaks (ENCODE accession ENCSR000BJK) as Topic-48. With this fine-tuned model, the explainability of the entire *TERT* promoter increases to 68% (Fig. 5B), and both *TERT* mutations are identified (Fig. 5C–E).

This provides us with a model that can potentially identify other functional gains or losses of GABPA binding sites. In fact, 43 ASCAVs across the 10 lines show a higher Topic-48 delta score than the known *TERT* promoter mutations. Thirteen of these are observed in other cancers (listed in COSMIC), and five are located in the promoter of a gene with ASE (Fig. 5D; Supplemental Fig. S15; Supplemental Table S11). Thus, the augmented DeepMEL2 model can be used to interpret *cis*-regulatory variation in melanoma genomes, with awareness of the MEL and MES enhancer code, the proximal promoter code, and *cis*-regulatory elements used by ETS-family members (Fig. 5F–H).

## Discussion

Functional variants affecting crucial genes and pathways are underpinning the fitness advantages of cancer cells. Such mutations may be found by recurrence analysis across patients and even across cancer types, at least in the coding fraction of the genome (Bailey et al. 2018). In the noncoding genome, however, this approach typically breaks down (Melton et al. 2015; Zhang et al. 2018), and *TERT* promoter mutations are a notable exception (Horn et al. 2013; Huang et al. 2013). Overall, noncoding mutations tend not to affect the same nucleotide or the same enhancer across samples. A recent large-scale and comprehensive whole-genome pan-cancer study from the ICGC-TCGA PCAWG consortium identified only 30 regionally recurrent *cis*-regulatory changes (Zhu et al. 2020). One reason for this might be the complex nature of gene regulation: Often multiple enhancers are brought into close proximity of a promoter to initiate transcription, and redundancy or cooperativity of these enhancers remains difficult to disentangle (Gasperini et al. 2020).

Here we address the challenge of identifying functional noncoding variants by focusing on allelic imbalances in chromatin accessibility and by linking those to changes in the enhancer sequence using an explainable AI model. Then, by exploiting phased genomes, we further link these potentially causal enhancer changes to allele-specific gene expression, TF binding, and histone acetylation.

Earlier work has shown that sample-matched epigenomic and transcriptomic data are instrumental to obtain a functional readout for genomic alterations (Stevenson et al. 2013; Castel et al. 2015; Chen et al. 2016). An intuitive approach to further establish causality is to assess how these variants affect the binding of TFs. Indeed, this strategy has previously been applied in several studies using PWMs to explain the impact of allele-specific noncoding variants (Deplancke et al. 2016). However, as PWM scoring requires stringent thresholds to limit the number of false-positive predictions, these models typically have low sensitivity (e.g., 3.3%–6.2% explainable variants) (Degner et al. 2012; Maurano et al. 2015; Chen et al. 2016; Tehranchi et al. 2019). More sophisticated machine-learning methods have been developed that can overcome these limitations. Models including support vector machines (Ghandi et al. 2014; Svetlichnyy et al. 2015) and neural networks (Alipanahi et al. 2015; Zhou and Troyanskaya 2015; Kelley et al. 2016; Liu et al. 2018) can be trained on enhancer sequences and used to predict the impact of mutations. In the context of allele-specific variant interpretation in normal genomes, Banovich et al. (2018) developed OrbWeaver, a four-layered neural network with log-transformed PWMs of 1320 TFs as the first layer. OrbWeaver was used to predict features of accessible chromatin in induced pluripotent stem cells and successfully captures the effect of chromatin accessibility QTL in a cell type–specific manner. In another study, Hoffman et al. (2019) developed DeepFIGV using DNase-seq and histone modification data from 75 lymphoblastoid cell lines and used it to predict ASB in an independent set of TF ChIP-seq data.

In addition to normal genomes, deep learning models have also been used to understand noncoding mutations in a disease context, such as autism spectrum disorders (Zhou et al. 2019) and pancreatic cancer (Feigin et al. 2017). These studies trained models on regulatory features from a diverse set of tissues and cell types profiled by the ENCODE and Roadmap Epigenomics projects. Although broadly applicable, this approach might limit model performance as regulatory activity is context dependent (The ENCODE Project Consortium 2012). Indeed, models trained on cell type–specific enhancers have been shown to yield better predictions (Banovich et al. 2018; Minnoye et al. 2020). The model we developed here is also context dependent and captures regulatory information from different melanoma cell states. The mesenchymal-like melanoma state, with a dominant role for AP-1, is shared with other cancer types (Baron et al. 2020) and is therefore well represented within ENCODE and other resources. As such, models trained on these large compendia (e.g., DeepSEA and Basset) can effectively identify AP-1 motif gains and losses. The melanocytic cell state, on the other hand, is less well represented. As a result, DeepMEL2 achieves a higher accuracy for all melanocytic samples than do DeepSEA and Basset.

We combined three components that improve the efficiency to detect enhancer mutations. First, we performed linked-read WGS on pure cancer cells (avoiding normal cell admixture); second, we incorporated matched ATAC-seq and RNA-seq data; and, third, we developed a context-dependent deep learning model, DeepMEL2. The use of pure cancer samples allowed us to accurately correct allelic signals in ATAC-seq, ChIP-seq, and RNA-seq data for genomic copy number alterations, leading to robust inference of functional allelic imbalances (Rozowsky et al. 2011; Chen et al. 2016; de Santiago et al. 2017).

By using DeepMEL2, 10%–16% of ASCAVs per MM line can be explained by changes in the *cis*-regulatory code. A sizable fraction of these were attributed to gains or losses of AP-1 binding sites, particularly in mesenchymal enhancers, which, in turn, could be confirmed by assaying ASB of the AP-1 family members JUN and FOSL1. We found that in melanocytic enhancers, gains and losses of SOX10 binding sites were most commonly linked with allele-specific chromatin accessibility. Although a large fraction of these events influences binding of a TF, without any other observed consequences, a subset does impact enhancer activity and is associated with changes in gene expression.

Finally, we investigated how context-specific models such as DeepMEL2, trained on epigenomic data, can be fine-tuned to better understand underrepresented enhancer logic. By examining the recurrently mutated *TERT* promoter, we found that the DeepMEL2 model did not use ETS motifs to classify melanoma enhancers. This may be owing to ETS binding sites not discriminating between MEL and MES enhancers, whereas other sequence features were more informative to predict classes. We resolved this limitation by providing the model with specific ChIP-seq data as an additional label. The augmented model achieved high prediction accuracies on the entire *TERT* promoter, including the known oncogenic mutations.

Our study shows that deep learning models provide a powerful means to pinpoint functional noncoding variation in cancer genomes, which may translate into clinical benefits for future patients. However, our results also suggest that each cancer type may require its own “matched” deep learning model, trained on epigenomic data from the relevant cancer cell states. Whereas the current work uses patient-derived cell cultures to infer ASCAVs, our framework is in principle also applicable to data obtained from bulk tumor biopsies. We envision that in a clinical setting, (1) deep learning models can be trained on ATAC-seq data from pure cancer cell clusters, when single-cell ATAC-seq is applied to a cohort of biopsies; and (2) genomics methods for single-nucleotide variant and copy number calling take normal cell admixture into account. It is worth noting that both points have been successfully shown in the literature (Satpathy et al. 2019; Dentro et al. 2021).

In conclusion, we have shown that high-confidence *cis*-regulatory variants can be detected by directly comparing the alleles of a cancer genome and using specialized predictive and explainable deep learning models trained on corresponding epigenomics profiles. Our compilation of multiome melanoma data and a melanoma-specific deep learning model provides unique data sets and a novel framework for understanding the impact of noncoding variants. Our approach is applicable to pure samples of any cancer type and may contribute to the identification of *cis*-regulatory driver mutations.

## Methods

### Cell culture

The melanoma MM lines are derived from patient biopsies by the Laboratory of Oncology and Experimental Surgery (Prof. Dr. Ghanem Ghanem) at the Institut Jules Bordet, Brussels (Gembarska et al. 2012; Verfaillie et al. 2015; Wouters et al. 2020). For further details on culture conditions, see Supplemental Methods.

### Phased whole-genome library preparation and sequencing

The extraction of high-molecular-weight (HMW) genomic DNA (gDNA) and the subsequent preparation of the phased whole-genome libraries were performed using the Chromium instrument and the linked-reads genome kit v2 (10x Genomics), according to the manufacturer's protocol (Rev A). Experimental details are elaborated in Supplemental Methods. Genomic aberrations per samples was visualized using Circos (Krzywinski et al. 2009).

### Copy number analysis

Allele-specific copy number calls were generated using ASCAT v2.5.2 (Van Loo et al. 2010). The analyses are further detailed in the Supplemental Methods.

We also assessed our ability to infer ASCAVs having arisen pre- and post-copy number gains (Supplemental Fig. S4). By leveraging the WGS variant allele-frequency (VAF), and the total tumor copy number and sample purity estimates from ASCAT (ntot and ρ = 100%, respectively), we can compute the number of chromosome copies carrying a variant (mutation copy number, mcn) as mcn = |ntot × VAF/ρ|. In turn, this can be used to “time” variants with respect to copy number gains: If a variant arose on a chromosome before its duplication, it will be duplicated as well (mcn ≥ 2, an “early” variant). If it arose after, only one copy will be present (mcn = 1, a “late” variant). In regions with loss of heterozygosity or gains on both alleles, these two scenarios can be readily distinguished. Note that, in our case, early variants will include germline SNPs.

### Personalized genome construction

Indels and SNVs (as generated by GATK/longranger) were used to construct personalized genomes with CrossStitch (https://github.com/schatzlab/crossstitch). This procedure resulted in the generation of personalized reference sequences per haplotype in FASTA format as well as chain files that link reference genome to personalized genomes. To obtain chain files to link personalized genomes to the reference genome, we performed whole-genome alignment between personalized genomes and reference genome using BLAT. The analyses are further detailed in Supplemental Methods.

### Mutational signatures

We use the Bayesian approach SigFit (Gori and Baez-Ortega 2020) to estimate mutation signature exposures in our MM lines for those COSMIC v3 signatures that have previously been reported as active in melanoma (SBS1, -2, -5, -7a–d, -9, -13, -14, -36, -38, and -40). SigFit was run on all likely somatic variants, namely, called variants not present in the gnomAD (v3.0), as well as on the likely somatic ASCAVs.

### ATAC-seq library preparation and sequencing

ATAC-seq data were generated using the Omni-ATAC-seq technique as described previously (Corces et al. 2017). For further detais, see Supplemental Methods.

### ATAC-seq alignment to the reference and personalized genomes

We aimed at obtaining minimum 15 million usable reads per sample, and eventually achieved 65 million reads on average across 10 sequenced samples (Supplemental Table S2). Paired-end reads were mapped to the human genome (hg38) and sample-specific personalized genomes using Bowtie 2 with ‐‐very-sensitive option (v2.2.6). Mapped reads were sorted using SAMtools (v1.8) (Li et al. 2009), and duplicates were removed using Picard MarkDuplicates (v1.134). Reads were filtered by removing chromosome mitochondria reads and filtering for Q > 2 using SAMtools. Usable reads are defined as the number of reads retained after these filtering steps. We observed that the number of reads mapping to personalized genomes was slightly higher than the number of reads mapping to the reference genome (hg38), which has been reported previously for ChIP-seq data (Supplemental Table S2; Rozowsky et al. 2011; Mayba et al. 2014).

### ATAC-seq peak calling

Peaks from ATAC-seq data were called for reference mapped and personalized genome mapped data using MACS2 (v2.1.2) (Zhang et al. 2008) using the parameters -q 0.05, ‐‐nomodel, ‐‐call-summits, ‐‐shift -75 ‐‐keep-dup all, and ‐‐extsize 150. Summits for personalized genome mapped samples were lifted over to reference genome using liftOver. Summits were extended by 250 bp upstream and downstream using slopBed (BEDTools, v2.28.0), providing human chromosome sizes, and filtered for blacklisted regions of the reference genome (ENCSR636HFF). Per sample, we obtained reference mapped peaks, HAP1 mapped peaks, and HAP2 mapped peaks. To obtain a consolidated peak set per sample, we followed the strategy described previously described (Corces et al. 2018), as elaborated in Supplemental Methods.

### Identification of allele-specific events in ATAC-seq data

We have built a new allele-specific variant detection pipeline using the backbone of the AlleleSeq pipeline (Chen et al. 2016). We used Bowtie 2 (Langmead and Salzberg 2012) to map ATAC-seq reads to personalized genomes as described above. Next, we marked duplicate reads in each alignment using Picard. Then, we evaluated two alignment files (i.e., haplotype 1 mapped and haplotype 2 mapped) to identify the most likely origin of each read. Each read was evaluated iteratively using mapping quality (MapQ), CIGAR string, and XM tag (which reports the number of mismatches in the alignment). If the read had the same mapQ, same CIGAR string (or the same number of Ms), and same XM tag for both alignments, it was marked as commonly mapping. This step resulted in four BAM files: haplotype1.exclusive, haplotype2.exclusive, haplotype1.common, and haplotype2.common.

After identifying the source of each read, we filtered out duplicate reads. We also filtered out ambiguously mapping reads by evaluating the reads that map equally well to both haplotypes (i.e., reads in haplotype1.common and haplotype2.common alignment files) in the reference genome. We lifted these positions over to hg38 coordinates, and we discarded reads if they mapped to different locations. Next, we overlapped phased heterozygous variants obtained from whole-genome sequence data with consolidated peak set (as described above). The variant positions that overlapped with the peaks were lifted over to haplotype 1 and 2 coordinates, and allele counts were obtained using the *samtools mpileup* command with all four alignment files (allelic counts coming from common alignment files were compared, and no major differences were found). Then allelic counts over heterozygous sites were merged, and variants that had at least six reads were further processed for allele-specific accessibility analysis with the BaalChIP (de Santiago et al. 2017) package in R/Bioconductor (R Core Team 2019). Count tables containing number of reference and alternative supporting reads per variant, together with the allelic ratio of the same variant from the whole-genome sequence data, were provided to runBayes command of BaalChIP, and ASCAVs were identified. The remaining variants were defined as control variants.

Genomic annotations of both sets of variants were performed using ChIPseeker (Yu et al. 2015) with the UCSC hg38 knownGene table (TxDb.Hsapiens.UCSC.hg38.knownGene package in R/Bioconductor).

### Motif enrichment analysis with ASB events

ASCA and control variants per sample were overlapped with consolidated ATAC-seq peaks. Peaks that had multiple variants were filtered out if the allelic bias between variants was inconsistent. Allelic counts were used to determine preferred allele (i.e., allele that has the highest ATAC-seq signal). Peak sequences were extracted from the FASTA sequence of the preferred allele (using fastaFromBed command from BEDTools) (Quinlan and Hall 2010). The reference sequence for each variant was extracted from hg38 using the same command. For each peak, we calculated the CRM score for the preferred allele and the other allele (reference sequence) using a set of 22,000 PWMs (Janky et al. 2014) and calculated a “delt a CRM score” for each peak and for each motif (Fig. 1D). We evaluated the enrichment of the CRM delta's in the ATAC-seq peaks using a one-sided Fisher's exact test with a control set of 152,999 peaks containing non-ASCA variants. We performed enrichment analysis individually (per MM-line) and globally (across all MM-lines) (Fig. 2C; Supplemental Fig. S9). Haplotype-resolved ATAC-seq alignment figures were created with fluff (Georgiou and van Heeringen 2016).

### Identification of ASEVs

RNA-seq reads were mapped to the personalized genomes using Bowtie 2 (Langmead and Salzberg 2012) with the ‐‐*very-sensitive* option. We implemented the same postprocessing steps as in the ATAC-seq analysis; this included choosing the best alignment between two mappings based on mapping quality and number of mismatches, as well as removal of ambiguously mapping or duplicate reads. Next, we overlaid phased heterozygous variants obtained from the WGS data with the coding genome (hg38 CDS regions). Variant positions falling inside genes were lifted over to haplotype 1 and 2 coordinates, and allele counts were obtained using samtools mpileup. Then allelic counts over heterozygous sites were merged, and variants that had at least 10 reads were further processed for allele-specific expression variant analysis. To assess allele-specific expression in the presence of copy number changes, we used a beta-binomial model of the RNA-seq allele counts, informed by the WGS data. Briefly, for every variant, we obtain the posterior estimate Beta(1 + #A, 1 + #B) of the WGS B-allele frequency using a uniform Beta(1, 1) prior and a binomial likelihood to describe the WGS allelic read counts (#A and #B). This posterior BAF estimate is then used to perform a two-tailed beta-binomial test of the observed RNA-seq allele counts. Multiple testing correction was implemented with the Benjamini–Hochberg method, and variants with a FDR < 0.05 were reported as allele-specific expression variants.

### Identification of allele-specific variants in H3K27ac ChIP-seq data

ChIP-seq reads were mapped personalized genomes using Bowtie 2 (Langmead and Salzberg 2012) with the ‐‐*very-sensitive* option. We implemented the same pipeline as in ATAC-seq analysis for allele-specific variant detection.

### cisTopic analysis

To train DeepMEL2 on, we used cisTopic (González-Blas et al. 2019) to obtain sets of coaccessible regions as in previous work in which we trained DeepMEL (Minnoye et al. 2020). To be able to use cisTopic, single cells were simulated from bulk Omni-ATAC-seq data on the 30 human melanoma cell lines. Bootstrapping was used for the single-cell simulation, and 50 single cells were simulated for each melanoma line. Each single cell contains 50,000 random reads from its original bulk Omni-ATAC-seq data. Then, cisTopic was run on these simulated single cells (parameters: α = 50/T, β = 0.1, burn-in iterations = 500, recording iterations = 1000). The best model (47 topics) was selected according to log-likelihood value.

### The DeepMEL2 neural network

In previous work, we developed DeepMEL on 16 human ATAC-seq samples (Minnoye et al. 2020). Here, we developed an updated model, DeepMEL2, on 30 ATAC-seq samples. DeepMEL2 is, similarly to DeepMEL, a hybrid CNN-RNN deep learning enhancer classification model composed of convolutional, max pooling, time-distributed dense, bidirectional LSTM, as well as dense layers between input and output. The 128 convolutional filters of DeepMEL were initialized with random numbers, whereas in DeepMEL2, 283 of 300 filters are populated with JASPAR PWMs that are clustered for five taxonomic groups (Fornes et al. 2020). Number of filters is increased from 128 to 300 and filter size is increased from 20 to 30 in order to populate convolutional filters with JASPAR motif collection. The detailed model architecture is shown in Supplemental Table S12. DeepMEL2 is trained on melanoma-specific coaccessible region classes. It takes a 500-bp DNA sequence and predicts an output vector corresponding to binarized 47 topics. To evaluate its performance, auROC and auPR on training (%80), validation (%10), and test (%10) sets were calculated for each topic.

DeepMEL2 + GABPA model was trained on 48 classes. On top of 47 topics, we added a 48th class in which regions in input data were labeled as 1 if it overlaps with GABPA ChIP-seq peaks (ENCSR000BJK). The same architecture that was used to train DeepMEL2 to was used for DeepMEL2 + GABPA

### Scoring enhancers and ASCAVs with DeepMEL2

To score ASCAVs, we perturbed the 500-bp ATAC-seq peaks by doing a single-nucleotide change according to variants coming from two alleles. We calculated delta prediction score for each of the ASCAVs and the control variants for each of the classes. Then, we evaluated the delta prediction scores for each class to identify the fraction of explainable ASCAVs. We used a one-sided Fisher's exact test with a control set of non-ASCA variants at 5% false-positive rate.

To compare different models, the maximum delta score for each variant was calculated.

### Calculating the contribution of each nucleotide to the final output

We initialized DeepExplainer (Lundberg and Lee 2017) with randomly selected sequences (500) and calculated the importance scores of the sequence of interest with respect to any of the 47 classes. We multiplied this importance score by the one-hot encoded matrix of the sequence. Finally, we visualized the sequence by adjusting the nucleotide heights based on their importance score, similar to earlier work (Shrikumar et al. 2019).

### In silico saturation mutagenesis

For a 500-bp sequence, we generated mutated sequences by changing each single nucleotide into the three other possible nucleotides. We scored the initial sequence without mutations, as well as all 1500 generated sequences with DeepMEL2, and calculated the delta prediction score for each class and for each mutation by comparing the final prediction relative to prediction for the initial sequence.

### Luciferase assays

The 501-bp regions, surrounded by 20-bp flanking adaptors, were synthesized (TWIST Bioscience) and then individually cloned in a pGL4.23 plasmid. Luciferase activity in MM057 was measured using the dual-Luciferase reporter assay system (Promega). Experimental details are elaborated in the Supplemental Methods.

### AP1 ChIP-seq library preparation and sequencing

The melanoma MM lines were grown to ∼85% confluence, and a total of 20 million cells per ChIP sample was collected. ChIP samples were prepared following the “Myers Laboratory ChIP-seq Protocol v011014,” using the following antibodies at a concentration of 5 µg per ChIP: FOS (c-Fos; sc-166940 X, Santa Cruz Biotechnology), FOSL1 (Fra-1; sc-376148, Santa Cruz Biotechnology), JUN (c-Jun; sc-74543 X, Santa Cruz Biotechnology), JUNB (Jun-B; sc-8051 X, Santa Cruz Biotechnology). For experimental details, see Supplemental Methods.

### Analysis of AP1 ChIP-seq data

Sequence reads were mapped to human reference genome (hg38) using Bowtie 2 with the ‐‐very-sensitive option (v2.2.6). Mapped reads were sorted using SAMtools (v1.8), and duplicates were removed using Picard MarkDuplicates (v1.134). Reads were filtered for mapping quality of 30 (MAPQ > 30) using SAMtools. We implemented the same pipeline as in ATAC-seq analysis for allele-specific variant detection.

### Publicly available data used in this work

RNA-seq data and H3K27ac ChIP-seq (data for A375, MM001, MM011, MM029, MM031, MM047, MM057, MM074, MM087, and MM099) were downloaded from the NCBI Gene Expression Omnibus (GEO; https://www.ncbi.nlm.nih.gov/geo/) under accession number GSE60666 (Verfaillie et al. 2015).

## Data access

All raw and processed sequencing data, except the WGS data, generated in this study have been submitted to the NCBI Gene Expression Omnibus (GEO; https://www.ncbi.nlm.nih.gov/geo/) under accession numbers GSE134432, GSE142238, and GSE159965. Genome sequencing data have been submitted to the European Genome-phenome Archive (EGA; http://www.ebi.ac.uk/ega/) under accession number EGAS00001004136. The code for the analysis of the WGS data and detection of ASCAV is available at GitHub (https://github.com/aertslab/AS_variant_pipeline) and as Supplemental Code. The DeepMEL2 and DeepMEL2_GABPA models are available from Kipoi (http://kipoi.org/models/DeepMEL/). The Jupyter notebooks to train DeepMEL and DeepMEL2 are available at GitHub (https://github.com/aertslab/DeepMEL), and the notebooks to train DeepMEL2 are provided as Supplemental Code.

## Supplementary Material

Supplemental Material
