# Supplementary material for: Interpretation of allele-specific chromatin accessibility using cell state–aware deep learning
Source: Genome Res. 2021 Jun;31(6):1082–96. doi: 10.1101/gr.260851.120 (PMC8168584; doi:10.1101/gr.260851.120)
Supplement: Supplemental Material [file supp_31_6_1082__DC1.html]

Interpretation of allele-specific chromatin accessibility using cell state–aware deep learning — Supplemental Material 

# Interpretation of allele-specific chromatin accessibility using cell state–aware deep learning

## Supplemental Material

- Supplemental\_Code.zip
- Supplementary\_Tables.zip
- Supplemental\_Figures.pdf
- Supplemental\_Methods.pdf
