# Supplementary material for: Interpretation of allele-specific chromatin accessibility using cell state–aware deep learning
Source: Genome Res. 2021 Jun;31(6):1082–96. doi: 10.1101/gr.260851.120 (PMC8168584; doi:10.1101/gr.260851.120)
Supplement: Supplemental Material [file supp_gr.260851.120_Supplemental_Code.zip › Supplemental_Code/DeepMEL2_scoring_and_deepexplainer.html]

DeepMEL2\_scoring\_and\_deepexplainer


# Notebook to perform prediction and plotting DeepExplainer by using DeepMEL2¶

#### Following codes in this notebook were run using conda environments¶

#### Here are the used packages and their version¶

In [ ]:

```
#%%bash
##cpu
# conda create --name DeepMEL2_conda_env_cpu python=3.6 tensorflow=1.14.0 keras=2.2.4
# conda activate DeepMEL2_conda_env_cpu
# conda install numpy=1.16.2 matplotlib=3.1.1 shap=0.29.3 ipykernel=5.1.2

# #gpu
# conda create --name DeepMEL2_conda_env_gpu python=3.6 tensorflow-gpu=1.14.0 keras-gpu=2.2.4
# conda activate DeepMEL2_conda_env_gpu
# conda install numpy=1.16.2  matplotlib=3.1.1 shap=0.29.3 ipykernel=5.1.2
```

#### Loading necessary packages¶

In [ ]:

```
from __future__ import absolute_import
from __future__ import division
from __future__ import print_function
import tempfile, sys, os
from array import *
sys.path.insert(0, os.path.abspath('..'))

import keras
from keras.models import Sequential, Model
from keras.models import model_from_json
from keras.layers import Dense, Dropout, Flatten, Activation, Bidirectional, Concatenate
from keras.layers.convolutional import Conv1D, MaxPooling1D
from keras.layers.recurrent import LSTM
from keras.utils import to_categorical, plot_model
from keras.callbacks import ModelCheckpoint
from keras import backend as K
K.set_learning_phase(False)

import shap
import shap.explainers.deep.deep_tf

import matplotlib
import matplotlib.pyplot as plt
import numpy as np
import sklearn
from sklearn.utils import shuffle
```

#### Defining necessary functitons¶

In [ ]:

```
def plot_a(ax, base, left_edge, height, color):
    a_polygon_coords = [
        np.array([
           [0.0, 0.0],
           [0.5, 1.0],
           [0.5, 0.8],
           [0.2, 0.0],
        ]),
        np.array([
           [1.0, 0.0],
           [0.5, 1.0],
           [0.5, 0.8],
           [0.8, 0.0],
        ]),
        np.array([
           [0.225, 0.45],
           [0.775, 0.45],
           [0.85, 0.3],
           [0.15, 0.3],
        ])
    ]
    for polygon_coords in a_polygon_coords:
        ax.add_patch(matplotlib.patches.Polygon((np.array([1,height])[None,:]*polygon_coords
                                                 + np.array([left_edge,base])[None,:]),
                                                facecolor=color, edgecolor=color))

def plot_c(ax, base, left_edge, height, color):
    ax.add_patch(matplotlib.patches.Ellipse(xy=[left_edge+0.65, base+0.5*height], width=1.3, height=height,
                                            facecolor=color, edgecolor=color))
    ax.add_patch(matplotlib.patches.Ellipse(xy=[left_edge+0.65, base+0.5*height], width=0.7*1.3, height=0.7*height,
                                            facecolor='white', edgecolor='white'))
    ax.add_patch(matplotlib.patches.Rectangle(xy=[left_edge+1, base], width=1.0, height=height,
                                            facecolor='white', edgecolor='white', fill=True))

def plot_g(ax, base, left_edge, height, color):
    ax.add_patch(matplotlib.patches.Ellipse(xy=[left_edge+0.65, base+0.5*height], width=1.3, height=height,
                                            facecolor=color, edgecolor=color))
    ax.add_patch(matplotlib.patches.Ellipse(xy=[left_edge+0.65, base+0.5*height], width=0.7*1.3, height=0.7*height,
                                            facecolor='white', edgecolor='white'))
    ax.add_patch(matplotlib.patches.Rectangle(xy=[left_edge+1, base], width=1.0, height=height,
                                            facecolor='white', edgecolor='white', fill=True))
    ax.add_patch(matplotlib.patches.Rectangle(xy=[left_edge+0.825, base+0.085*height], width=0.174, height=0.415*height,
                                            facecolor=color, edgecolor=color, fill=True))
    ax.add_patch(matplotlib.patches.Rectangle(xy=[left_edge+0.625, base+0.35*height], width=0.374, height=0.15*height,
                                            facecolor=color, edgecolor=color, fill=True))

def plot_t(ax, base, left_edge, height, color):
    ax.add_patch(matplotlib.patches.Rectangle(xy=[left_edge+0.4, base],
                  width=0.2, height=height, facecolor=color, edgecolor=color, fill=True))
    ax.add_patch(matplotlib.patches.Rectangle(xy=[left_edge, base+0.8*height],
                  width=1.0, height=0.2*height, facecolor=color, edgecolor=color, fill=True))

default_colors = {0:'green', 1:'blue', 2:'orange', 3:'red'}
default_plot_funcs = {0:plot_a, 1:plot_c, 2:plot_g, 3:plot_t}
def plot_weights_given_ax(ax, array,
                 height_padding_factor,
                 length_padding,
                 subticks_frequency,
                 highlight,
                 colors=default_colors,
                 plot_funcs=default_plot_funcs):
    if len(array.shape)==3:
        array = np.squeeze(array)
    assert len(array.shape)==2, array.shape
    if (array.shape[0]==4 and array.shape[1] != 4):
        array = array.transpose(1,0)
    assert array.shape[1]==4
    max_pos_height = 0.0
    min_neg_height = 0.0
    heights_at_positions = []
    depths_at_positions = []
    for i in range(array.shape[0]):
        acgt_vals = sorted(enumerate(array[i,:]), key=lambda x: abs(x[1]))
        positive_height_so_far = 0.0
        negative_height_so_far = 0.0
        for letter in acgt_vals:
            plot_func = plot_funcs[letter[0]]
            color=colors[letter[0]]
            if (letter[1] > 0):
                height_so_far = positive_height_so_far
                positive_height_so_far += letter[1]                
            else:
                height_so_far = negative_height_so_far
                negative_height_so_far += letter[1]
            plot_func(ax=ax, base=height_so_far, left_edge=i, height=letter[1], color=color)
        max_pos_height = max(max_pos_height, positive_height_so_far)
        min_neg_height = min(min_neg_height, negative_height_so_far)
        heights_at_positions.append(positive_height_so_far)
        depths_at_positions.append(negative_height_so_far)
    for color in highlight:
        for start_pos, end_pos in highlight[color]:
            assert start_pos >= 0.0 and end_pos <= array.shape[0]
            min_depth = np.min(depths_at_positions[start_pos:end_pos])
            max_height = np.max(heights_at_positions[start_pos:end_pos])
            ax.add_patch(
                matplotlib.patches.Rectangle(xy=[start_pos,min_depth],
                    width=end_pos-start_pos,
                    height=max_height-min_depth,
                    edgecolor=color, fill=False))
            
    ax.set_xlim(-length_padding, array.shape[0]+length_padding)
    ax.xaxis.set_ticks(np.arange(0.0, array.shape[0]+1, subticks_frequency))
    height_padding = max(abs(min_neg_height)*(height_padding_factor),
                         abs(max_pos_height)*(height_padding_factor))
    ax.set_ylim(min_neg_height-height_padding, max_pos_height+height_padding)
    return ax

def plot_weights_modified(array, fig, n,n1,n2, title='', ylab='',
                              figsize=(20,2),
                 height_padding_factor=0.2,
                 length_padding=1.0,
                 subticks_frequency=20,
                 colors=default_colors,
                 plot_funcs=default_plot_funcs,
                 highlight={}):
    ax = fig.add_subplot(n,n1,n2) 
    ax.set_title(title)
    ax.set_ylabel(ylab)
    y = plot_weights_given_ax(ax=ax, array=array,
        height_padding_factor=height_padding_factor,
        length_padding=length_padding,
        subticks_frequency=subticks_frequency,
        colors=colors,
        plot_funcs=plot_funcs,
        highlight=highlight)
    return fig,ax

def json_hdf5_to_model(json_filename, hdf5_filename):  
    with open(json_filename, 'r') as f:
        model = model_from_json(f.read())
    model.load_weights(hdf5_filename)
    return model

def readfile(filename):
    ids = []
    ids_d = {}
    seqs = {}
    classes = {}
    f = open(filename, 'r')
    lines = f.readlines()
    f.close()
    seq = []
    for line in lines:
        if line[0] == '>':
            ids.append(line[1:].rstrip('\n'))
            if line[1:].rstrip('\n').split('_')[0] not in seqs:
                seqs[line[1:].rstrip('\n').split('_')[0]] = []
            if line[1:].rstrip('\n').split('_')[0] not in ids_d:
                ids_d[line[1:].rstrip('\n').split('_')[0]] = line[1:].rstrip('\n').split('_')[0]
            if line[1:].rstrip('\n').split('_')[0] not in classes:
                classes[line[1:].rstrip('\n').split('_')[0]] = np.zeros(NUM_CLASSES)
            classes[line[1:].rstrip('\n').split('_')[0]][int(line[1:].rstrip('\n').split('_')[1])-1] = 1        
            if seq != []: seqs[ids[-2].split('_')[0]]= ("".join(seq))
            seq = []
        else:
            seq.append(line.rstrip('\n').upper())
    if seq != []:
        seqs[ids[-1].split('_')[0]]=("".join(seq))

    return ids,ids_d,seqs,classes

def readfile_wolabel(filename):
    ids = []
    ids_d = {}
    seqs = {}
    f = open(filename, 'r')
    lines = f.readlines()
    f.close()
    seq = []
    for line in lines:
        if line[0] == '>':
            ids.append(line[1:].rstrip('\n'))
            if line[1:].rstrip('\n') not in seqs:
                seqs[line[1:].rstrip('\n')] = []
            if line[1:].rstrip('\n') not in ids_d:
                ids_d[line[1:].rstrip('\n')] = line[1:].rstrip('\n')    
            if seq != []: seqs[ids[-2]]= ("".join(seq))
            seq = []
        else:
            seq.append(line.rstrip('\n').upper())
    if seq != []:
        seqs[ids[-1]]=("".join(seq))

    return ids,ids_d,seqs


def one_hot_encode_along_row_axis(sequence):
    to_return = np.zeros((1,len(sequence),4), dtype=np.int8)
    seq_to_one_hot_fill_in_array(zeros_array=to_return[0],
                                 sequence=sequence, one_hot_axis=1)
    return to_return


def seq_to_one_hot_fill_in_array(zeros_array, sequence, one_hot_axis):
    assert one_hot_axis==0 or one_hot_axis==1
    if (one_hot_axis==0):
        assert zeros_array.shape[1] == len(sequence)
    elif (one_hot_axis==1): 
        assert zeros_array.shape[0] == len(sequence)
    for (i,char) in enumerate(sequence):
        if (char=="A" or char=="a"):
            char_idx = 0
        elif (char=="C" or char=="c"):
            char_idx = 1
        elif (char=="G" or char=="g"):
            char_idx = 2
        elif (char=="T" or char=="t"):
            char_idx = 3
        elif (char=="N" or char=="n"):
            continue #leave that pos as all 0's
        else:
            raise RuntimeError("Unsupported character: "+str(char))
        if (one_hot_axis==0):
            zeros_array[char_idx,i] = 1
        elif (one_hot_axis==1):
            zeros_array[i,char_idx] = 1
            
def path_to_id_dict(path):
    filename = path
    ids, ids_d, seqs, classes = readfile_wolabel(filename)
    result = {'ids':ids, 'ids_d':ids_d, 'seqs':seqs, 'classes':classes, }
    return result

def path_to_X_id_dict(path):
    filename = path
    ids, ids_d, seqs = readfile_wolabel(filename)
    X = np.array([one_hot_encode_along_row_axis(seqs[id]) for id in ids_d]).squeeze(axis=1)
    X_rc = [X,  X[:,::-1,::-1]]
    result = {'ids':ids, 'ids_d':ids_d, 'seqs':seqs, 'X':X, 'X_rc':X_rc }
    return result

def path_to_X_id_dict_label(path):
    train_filename = path   
    train_ids,train_ids_d, train_seqs, train_classes = readfile(train_filename)
    X = np.array([one_hot_encode_along_row_axis(train_seqs[id]) for id in train_ids_d]).squeeze(axis=1)
    y = np.array([train_classes[id] for id in train_ids_d])

    y = y[:,selected_classes]
    X = X[y.sum(axis=1)>0]
    ids = np.array([id for id in train_ids_d])
    ids = ids[y.sum(axis=1)>0]
    y = y[y.sum(axis=1)>0]
    X_rc = [X,  X[:,::-1,::-1]]
    
    X_single=X[y.sum(axis=1)==1]
    y_single=y[y.sum(axis=1)==1]
    ids_single=ids[y.sum(axis=1)==1]
    
    result = {'ids':train_ids, 'ids_d':train_ids_d, 'seqs':train_seqs, 'classes':train_classes, 'X':X, 'X_rc':X_rc, 'y':y ,"y_single":y_single}
    return result   

def loc_to_model_loss(loc):
    return json_hdf5_to_model(loc + 'model.json', loc + 'model_best_loss.hdf5')

def shuffle_label(label):
    for i in range(len(label.T)):
        label.T[i] = shuffle(label.T[i])
    return label

def calculate_roc_pr(score, label):
    output = np.zeros((len(label.T), 2))
    for i in range(len(label.T)):
        roc_ = roc_auc_score(label.T[i], score.T[i])
        pr_ = average_precision_score(label.T[i], score.T[i])
        output[i] = [roc_, pr_]
    return output
```

#### Preparing the input data¶

In [ ]:

```
seq_shape = [500,4]
NUM_CLASSES=47
selected_classes = np.array(list(range(NUM_CLASSES)))

train_filename = '/results/summits_to_topics.fa'
train_ids,train_ids_d, train_seqs, train_classes = readfile(train_filename)
X = np.array([one_hot_encode_along_row_axis(train_seqs[id]) for id in train_ids_d]).squeeze(axis=1)
ids = np.array([id for id in train_ids_d])
y = np.array([train_classes[id] for id in train_ids_d])
y = y[:,selected_classes]

X = X[y.sum(axis=1)>0]
ids = ids[y.sum(axis=1)>0]
y = y[y.sum(axis=1)>0]
X_rc = [X,  X[:,::-1,::-1]]
```

#### Loading DeepMEL2 and initialising DeepExplainer¶

In [ ]:

```
np.random.seed(seed=777)
trained_model = loc_to_model_loss('/results/')
rn=np.random.choice(X_rc[0].shape[0], 500, replace=False)
explainer = shap.DeepExplainer((trained_model.inputs, trained_model.layers[-1].output), [X_rc[0][rn],X_rc[1][rn]] )
```

#### Performing prediction on one of the input sequences¶

In [ ]:

```
region_name = 'id_of_the_region'
prediction=trained_model.predict([X_rc[0][ids==region_name],X_rc[1][ids==region_name]])[0]
plt.plot(prediction,'--',marker="o")
plt.title(region_name)
plt.ylabel("Predictions")
plt.xlabel("Topics")
_ = plt.xticks(range(len(selected_classes)),range(1,len(selected_classes)+1))
```

#### Plotting DeepExplainer and in silico saturation mutagenesis on one of the input sequences¶

In [ ]:

```
region_name = 'id_of_the_region'
shap_values, indexes = explainer.shap_values([X_rc[0][ids==region_name],X_rc[1][ids==region_name]], ranked_outputs=1)

ntrack=3
fig = plt.figure(figsize=(80,10))
_, ax1 =plot_weights_modified(shap_values[0][0][0]*X_rc[0][ids==region_name],fig,ntrack,1,1,title=region_name, subticks_frequency=10,ylab="FORWARD\nDeepExplainer")
_, ax2 =plot_weights_modified((shap_values[0][1][0]*X_rc[1][ids==region_name])[0][::-1,:],fig,ntrack,1,2,subticks_frequency=10,ylab="REVERSE\nDeepExplainer")
ax1.set_ylim([np.min([ax1.get_ylim()[0],ax2.get_ylim()[0] ]) , np.max([ax1.get_ylim()[1],ax2.get_ylim()[1] ])])
ax2.set_ylim([np.min([ax1.get_ylim()[0],ax2.get_ylim()[0] ]) , np.max([ax1.get_ylim()[1],ax2.get_ylim()[1] ])])

arrr_A = np.zeros((len(selected_classes),X_rc[0].shape[1]))
arrr_C = np.zeros((len(selected_classes),X_rc[0].shape[1]))
arrr_G = np.zeros((len(selected_classes),X_rc[0].shape[1]))
arrr_T = np.zeros((len(selected_classes),X_rc[0].shape[1]))
new_X = np.copy(X_rc[0][ids==region_name])
real_score=trained_model.predict([new_X,new_X[:,::-1,::-1]])[0]
for mutloc in range(seq_shape[0]):     
    new_X_= np.copy(new_X)
    new_X_[0][mutloc,:] = np.array([1, 0, 0, 0], dtype='int8')
    arrr_A[:,mutloc]=(real_score - trained_model.predict([new_X_,new_X_[:,::-1,::-1]])[0])
    new_X_[0][mutloc,:] = np.array([0, 1, 0, 0], dtype='int8')
    arrr_C[:,mutloc]=(real_score - trained_model.predict([new_X_,new_X_[:,::-1,::-1]])[0])
    new_X_[0][mutloc,:] = np.array([0, 0, 1, 0], dtype='int8')
    arrr_G[:,mutloc]=(real_score - trained_model.predict([new_X_,new_X_[:,::-1,::-1]])[0])
    new_X_[0][mutloc,:] = np.array([0, 0, 0, 1], dtype='int8')
    arrr_T[:,mutloc]=(real_score - trained_model.predict([new_X_,new_X_[:,::-1,::-1]])[0])
arrr_A[arrr_A==0]=None
arrr_C[arrr_C==0]=None
arrr_G[arrr_G==0]=None
arrr_T[arrr_T==0]=None

ax = fig.add_subplot(ntrack,1,3)
ax.scatter(range(X_rc[0].shape[1]),-1*arrr_A[indexes[0][0]],label='A',color='green')
ax.scatter(range(X_rc[0].shape[1]),-1*arrr_C[indexes[0][0]],label='C',color='blue')
ax.scatter(range(X_rc[0].shape[1]),-1*arrr_G[indexes[0][0]],label='G',color='orange')
ax.scatter(range(X_rc[0].shape[1]),-1*arrr_T[indexes[0][0]],label='T',color='red')
ax.legend()
ax.axhline(y=0,linestyle='--',color='gray')
ax.set_xlim((0,X_rc[0].shape[1])) 
_ = ax.set_xticks(np.arange(0, seq_shape[0]+1, 10))
```
