# Supplementary material for: Interpretation of allele-specific chromatin accessibility using cell state–aware deep learning
Source: Genome Res. 2021 Jun;31(6):1082–96. doi: 10.1101/gr.260851.120 (PMC8168584; doi:10.1101/gr.260851.120)
Supplement: Supplemental Material [file supp_gr.260851.120_Supplemental_Code.zip › Supplemental_Code/DeepMEL2_training.html]

DeepMEL2\_training


# Notebook to train DeepMEL2¶

#### Following codes in this notebook were run using conda environments¶

#### Here are the used packages and their version¶

In [ ]:

```
%%bash
#cpu
conda create --name DeepMEL2_conda_env_cpu python=3.6 tensorflow=1.14.0 keras=2.2.4
conda activate DeepMEL2_conda_env_cpu
conda install numpy=1.16.2 matplotlib=3.1.1 shap=0.29.3 ipykernel=5.1.2

#gpu
conda create --name DeepMEL2_conda_env_gpu python=3.6 tensorflow-gpu=1.14.0 keras-gpu=2.2.4
conda activate DeepMEL2_conda_env_gpu
conda install numpy=1.16.2  matplotlib=3.1.1 shap=0.29.3 ipykernel=5.1.2
```

#### Loading necessary packages¶

In [ ]:

```
import sys
import optparse
from array import *

import tensorflow
import numpy as np
import pickle

import matplotlib
import matplotlib.pyplot as plt

import sklearn
from sklearn.utils import class_weight, shuffle
from sklearn.model_selection import train_test_split
from sklearn.metrics import average_precision_score, roc_auc_score

from keras.models import Sequential
from keras.models import model_from_json
from keras.layers.core import Dense, Dropout, Activation, Flatten
from keras.layers.convolutional import Conv1D, MaxPooling1D
from keras.layers import Bidirectional, Concatenate, PReLU 
from keras.optimizers import RMSprop, Adam
from keras.callbacks import ModelCheckpoint, EarlyStopping
from keras import regularizers
from keras.layers.wrappers import Bidirectional, TimeDistributed
from keras.layers.recurrent import LSTM
from keras.layers import Layer, average, Input
from keras.models import Model
from keras.utils import plot_model
```

#### Defining necessary functitons¶

In [ ]:

```
def get_output(input_layer, hidden_layers):
    output = input_layer
    for hidden_layer in hidden_layers: 
        output = hidden_layer(output)
    return output

def build_model():
    forward_input = Input(shape=seq_shape)
    reverse_input = Input(shape=seq_shape)

    hidden_layers = [
        Conv1D(300, kernel_size=30, padding="valid", activation='relu', kernel_initializer='random_uniform'),
        MaxPooling1D(pool_size=15, strides=5, padding='valid'),
        Dropout(0.2),
        TimeDistributed(Dense(128, activation='relu')),
        Bidirectional(LSTM(128, dropout=0.1, recurrent_dropout=0.1, return_sequences=True)),
        Dropout(0.2),
        Flatten(),
        Dense(256, activation='relu'),
        Dropout(0.4),
        Dense(47, activation='sigmoid')]
    forward_output = get_output(forward_input, hidden_layers)     
    reverse_output = get_output(reverse_input, hidden_layers)
    output = average([forward_output, reverse_output])
    model = Model(input=[forward_input, reverse_input], output=output)
    
    f = open("/motif_file.pkl", "rb")
    motif_dict = pickle.load(f)
    f.close()
    conv_weights = model.layers[2].get_weights()
    for i,name in enumerate(motif_dict):
        conv_weights[0][:,:,i] = conv_weights[0][:, :, i] * 0.1
        conv_weights[0][int((30-len(motif_dict[name]))/2):int((30-len(motif_dict[name]))/2) + len(motif_dict[name]), :, i] = motif_dict[name]
    model.layers[2].set_weights(conv_weights)

    model.summary()
    model.compile(optimizer='adam', loss='binary_crossentropy', metrics=['accuracy'])
    return model

def readfile(filename):
    ids = []
    ids_d = {}
    seqs = {}
    classes = {}
    f = open(filename, 'r')
    lines = f.readlines()
    f.close()
    seq = []
    for line in lines:
        if line[0] == '>':
            ids.append(line[1:].rstrip('\n'))
            if line[1:].rstrip('\n').split('_')[0] not in seqs:
                seqs[line[1:].rstrip('\n').split('_')[0]] = []
            if line[1:].rstrip('\n').split('_')[0] not in ids_d:
                ids_d[line[1:].rstrip('\n').split('_')[0]] = line[1:].rstrip('\n').split('_')[0]
            if line[1:].rstrip('\n').split('_')[0] not in classes:
                classes[line[1:].rstrip('\n').split('_')[0]] = np.zeros(NUM_CLASSES)
            classes[line[1:].rstrip('\n').split('_')[0]][int(line[1:].rstrip('\n').split('_')[1])-1] = 1        
            if seq != []: seqs[ids[-2].split('_')[0]]= ("".join(seq))
            seq = []
        else:
            seq.append(line.rstrip('\n').upper())
    if seq != []:
        seqs[ids[-1].split('_')[0]]=("".join(seq))
    return ids,ids_d,seqs,classes

def one_hot_encode_along_row_axis(sequence):
    to_return = np.zeros((1,len(sequence),4), dtype=np.int8)
    seq_to_one_hot_fill_in_array(zeros_array=to_return[0],
                                 sequence=sequence, one_hot_axis=1)
    return to_return

def seq_to_one_hot_fill_in_array(zeros_array, sequence, one_hot_axis):
    assert one_hot_axis==0 or one_hot_axis==1
    if (one_hot_axis==0):
        assert zeros_array.shape[1] == len(sequence)
    elif (one_hot_axis==1): 
        assert zeros_array.shape[0] == len(sequence)
    for (i,char) in enumerate(sequence):
        if (char=="A" or char=="a"):
            char_idx = 0
        elif (char=="C" or char=="c"):
            char_idx = 1
        elif (char=="G" or char=="g"):
            char_idx = 2
        elif (char=="T" or char=="t"):
            char_idx = 3
        elif (char=="N" or char=="n"):
            continue
        else:
            raise RuntimeError("Unsupported character: "+str(char))
        if (one_hot_axis==0):
            zeros_array[char_idx,i] = 1
        elif (one_hot_axis==1):
            zeros_array[i,char_idx] = 1

def create_plots(history):
    plt.plot(history.history['acc'])
    plt.plot(history.history['val_acc'])
    plt.title('model accuracy')
    plt.ylabel('accuracy')
    plt.xlabel('epoch')
    plt.legend(['train', 'test'], loc='upper left')
    plt.savefig(foldername + 'accuracy.png')
    plt.clf()

    plt.plot(history.history['loss'])
    plt.plot(history.history['val_loss'])
    plt.title('model loss')
    plt.ylabel('loss')
    plt.xlabel('epoch')
    plt.legend(['train', 'test'], loc='upper left')
    plt.savefig(foldername + 'loss.png')
    plt.clf()
    
def json_hdf5_to_model(json_filename, hdf5_filename):  
    with open(json_filename, 'r') as f:
        model = model_from_json(f.read())
    model.load_weights(hdf5_filename)
    return model

def loc_to_model_loss(loc):
    return json_hdf5_to_model(loc + 'model.json', loc + 'model_best_loss.hdf5')

def shuffle_label(label):
    for i in range(len(label.T)):
        label.T[i] = shuffle(label.T[i])
    return label

def calculate_roc_pr(score, label):
    output = np.zeros((len(label.T), 2))
    for i in range(len(label.T)):
        roc_ = roc_auc_score(label.T[i], score.T[i])
        pr_ = average_precision_score(label.T[i], score.T[i])
        output[i] = [roc_, pr_]
    return output
```

#### Preparing the input data¶

In [ ]:

```
NUM_CLASSES = 47
selected_classes = np.array(list(range(NUM_CLASSES)))
SEQ_LEN = 500
SEQ_DIM = 4
seq_shape = (SEQ_LEN, SEQ_DIM)
EPOCH = 20
BATCH = 2048
PATIENCE = 5

foldername = '/results/'
train_filename = '/summits_to_topics_train.fa'
valid_filename = '/summits_to_topics_validation.fa'
test_filename = '/summits_to_topics_test.fa'

PATH_TO_SAVE_ARC = foldername + 'model.json'
PATH_TO_SAVE_BEST_LOST_WEIGHTS = foldername + 'model_best_loss'
PATH_TO_SAVE_BEST_ACC_WEIGHTS = foldername + 'model_best_acc'
PATH_TO_SAVE_END_WEIGHTS = foldername + 'model_end.hdf5'

print("Prepare input...")
train_ids, train_ids_d, train_seqs, train_classes = readfile(train_filename)
X_train = np.array([one_hot_encode_along_row_axis(train_seqs[id]) for id in train_ids_d]).squeeze(axis=1)
y_train = np.array([train_classes[id] for id in train_ids_d])
y_train = y_train[:,selected_classes]
X_train = X_train[y_train.sum(axis=1)>0]
y_train = y_train[y_train.sum(axis=1)>0]
X_train_rc = X_train[:,::-1,::-1]
train_data = [X_train, X_train_rc]

valid_ids, valid_ids_d, valid_seqs, valid_classes = readfile(valid_filename)
X_valid = np.array([one_hot_encode_along_row_axis(valid_seqs[id]) for id in valid_ids_d]).squeeze(axis=1)
y_valid = np.array([valid_classes[id] for id in valid_ids_d])
y_valid = y_valid[:,selected_classes]
X_valid = X_valid[y_valid.sum(axis=1)>0]
y_valid = y_valid[y_valid.sum(axis=1)>0]
X_valid_rc = X_valid[:,::-1,::-1]
valid_data = [X_valid, X_valid_rc]

test_ids, test_ids_d, test_seqs, test_classes = readfile(test_filename)
X_test = np.array([one_hot_encode_along_row_axis(test_seqs[id]) for id in test_ids_d]).squeeze(axis=1)
y_test = np.array([test_classes[id] for id in test_ids_d])
y_test = y_test[:,selected_classes]
X_test = X_test[y_test.sum(axis=1)>0]
y_test = y_test[y_test.sum(axis=1)>0]
X_test_rc = X_test[:,::-1,::-1]
test_data = [X_test, X_test_rc]
```

#### Training the model¶

In [ ]:

```
print("Compile model...")
model = build_model()

model_json = model.to_json()
with open(PATH_TO_SAVE_ARC, "w") as json_file:
    json_file.write(model_json)
print("Model architecture saved to..", PATH_TO_SAVE_ARC)

checkpoint1 = ModelCheckpoint(PATH_TO_SAVE_BEST_LOST_WEIGHTS+"_{epoch:02d}.hdf5", monitor='val_loss', verbose=1, save_best_only=True, mode='min')
checkpoint2 = ModelCheckpoint(PATH_TO_SAVE_BEST_ACC_WEIGHTS+"_{epoch:02d}.hdf5", monitor='val_acc', verbose=1, save_best_only=True, mode='max')
checkpoint3 = EarlyStopping(monitor='val_loss', patience=PATIENCE)
callbacks_list = [checkpoint1, checkpoint2, checkpoint3]

print("Train model...")
history = model.fit( train_data, y_train, nb_epoch=EPOCH, batch_size=BATCH, shuffle=True, validation_data=(valid_data, y_valid), verbose=1, callbacks=callbacks_list)
create_plots(history)
model.save_weights(PATH_TO_SAVE_END_WEIGHTS)
print("Model weights saved to..", PATH_TO_SAVE_END_WEIGHTS)
plot_model(model, to_file=foldername + 'model.png')

score, acc = model.evaluate(test_data, y_test, batch_size=BATCH)
print('Test score:', score)
print('Test accuracy:', acc)
```
