# Supplementary material for: Interpretation of allele-specific chromatin accessibility using cell state–aware deep learning
Source: Genome Res. 2021 Jun;31(6):1082–96. doi: 10.1101/gr.260851.120 (PMC8168584; doi:10.1101/gr.260851.120)
Supplement: Supplemental Material [file supp_gr.260851.120_Supplemental_Figures.pdf]

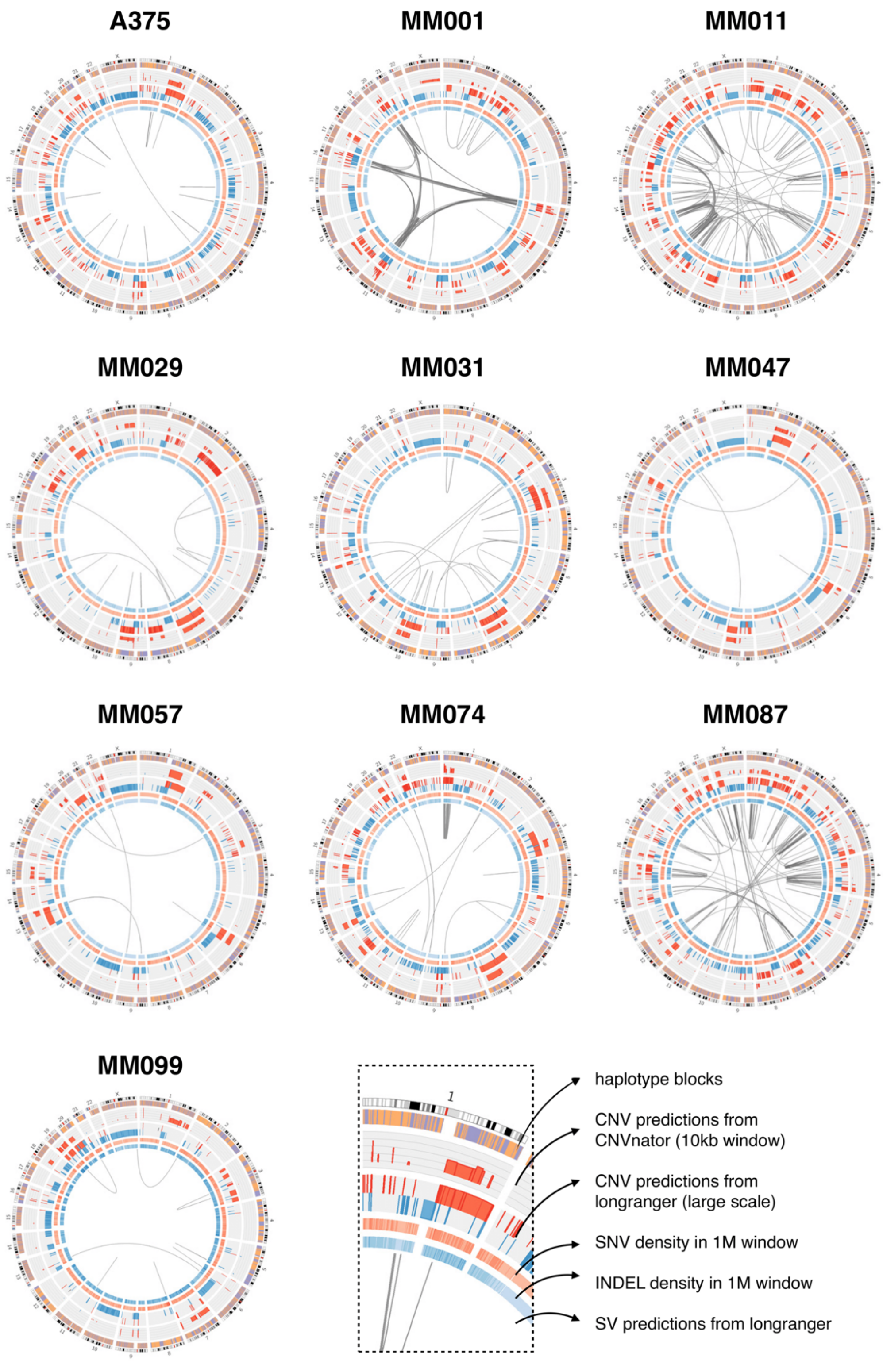

Supplemental Figure S1. Circos plots of 10 melanoma lines showing haplotype blocks, predictions of copy number alterations from two different prediction methods (CNVNator and longranger), SNV & INDEL density in 1Mb windows, and structural variant predictions.

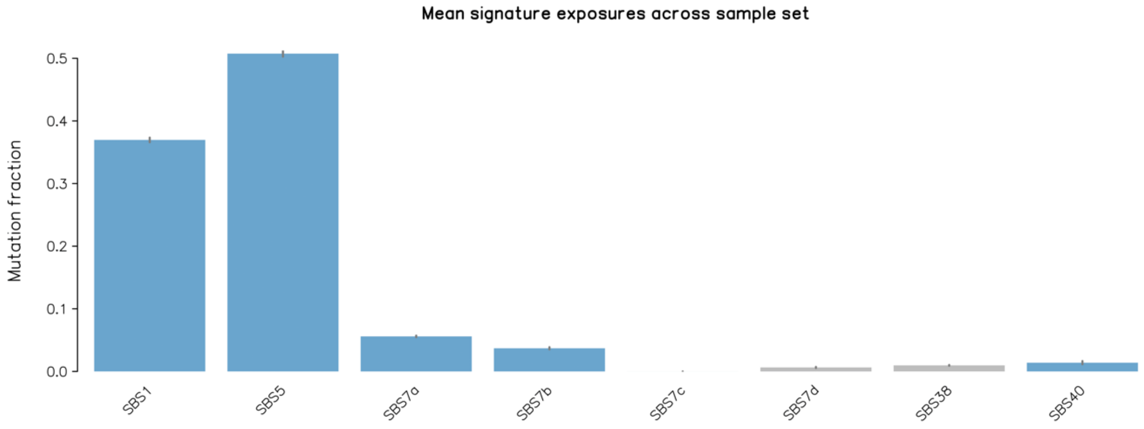
Mean signature exposures across sample set
Mutation fraction
0.5
0.4
0.3
0.2
0.1
0.0
SBS1
SBS5
SBS7a
SBS7b
SBS7c
SBS7d
SBS38
SBS40

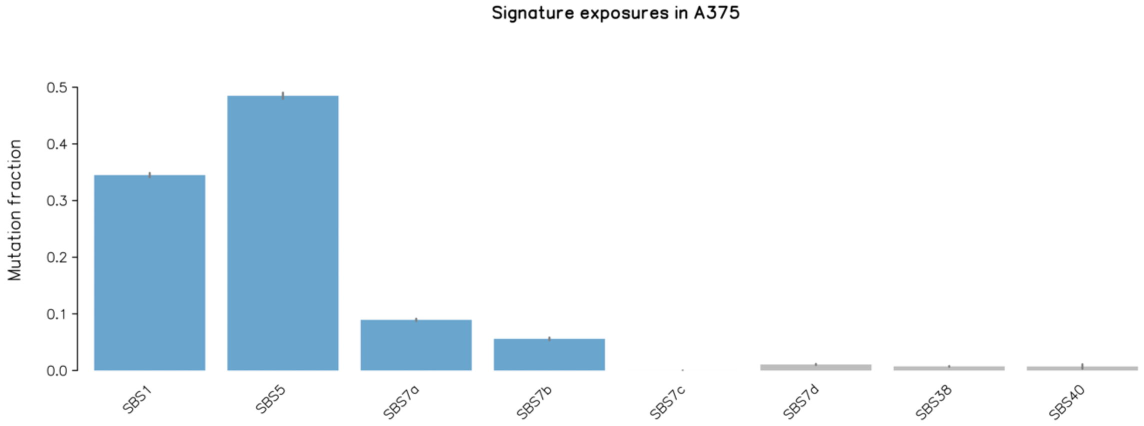
Signature exposures in A375
Mutation fraction
0.5
0.4
0.3
0.2
0.1
0.0
SBS1
SBS5
SBS7a
SBS7b
SBS7c
SBS7d
SBS38
SBS40

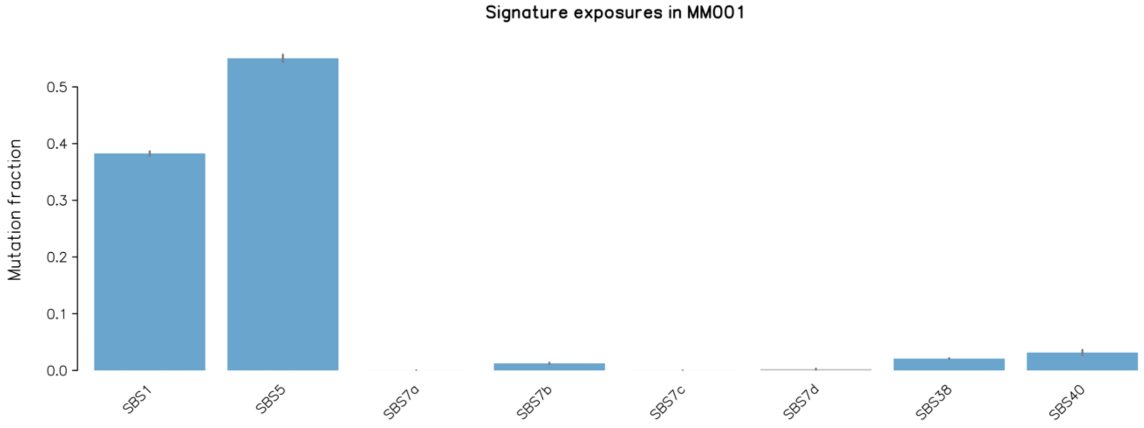
Signature exposures in MM001
Mutation fraction
0.5
0.4
0.3
0.2
0.1
0.0
SBS1
SBS5
SBS7a
SBS7b
SBS7c
SBS7d
SBS38
SBS40

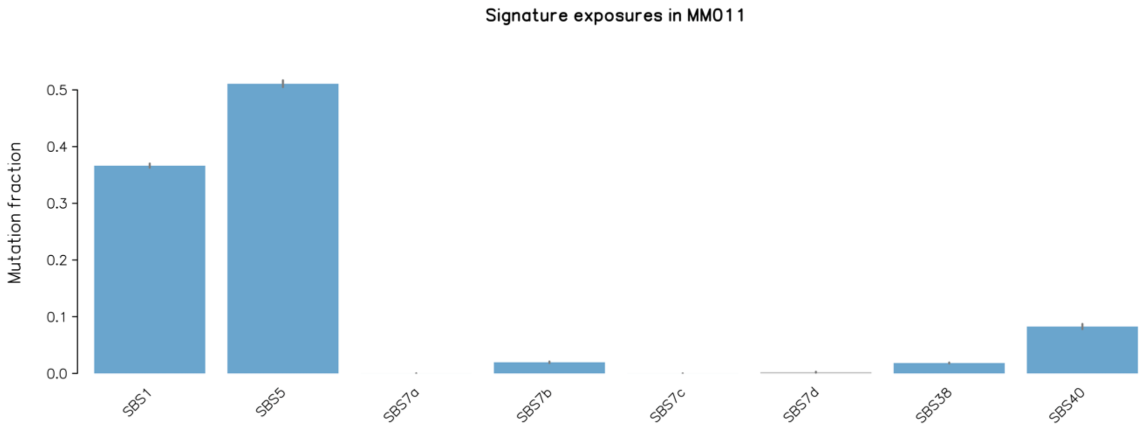
Signature exposures in MM011
Mutation fraction
0.5
0.4
0.3
0.2
0.1
0.0
SBS1
SBS5
SBS7a
SBS7b
SBS7c
SBS7d
SBS38
SBS40

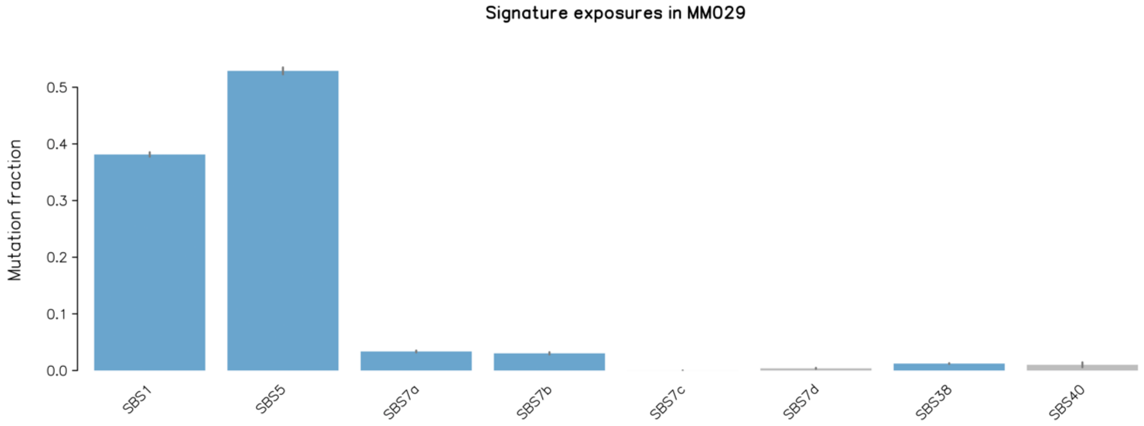
Signature exposures in MM029
Mutation fraction
0.5
0.4
0.3
0.2
0.1
0.0
SBS1
SBS5
SBS7a
SBS7b
SBS7c
SBS7d
SBS38
SBS40

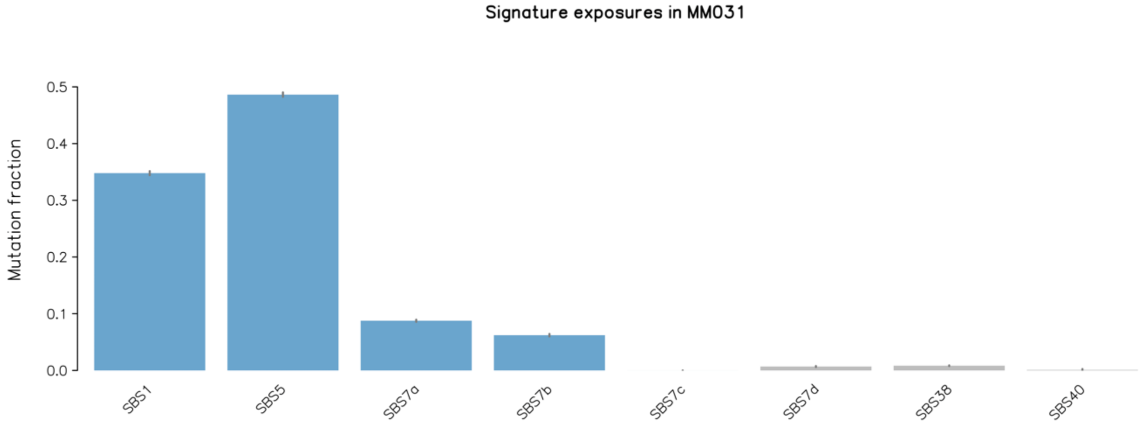
Signature exposures in MM031
Mutation fraction
0.5
0.4
0.3
0.2
0.1
0.0
SBS1
SBS5
SBS7a
SBS7b
SBS7c
SBS7d
SBS38
SBS40

Signature exposures in MM047

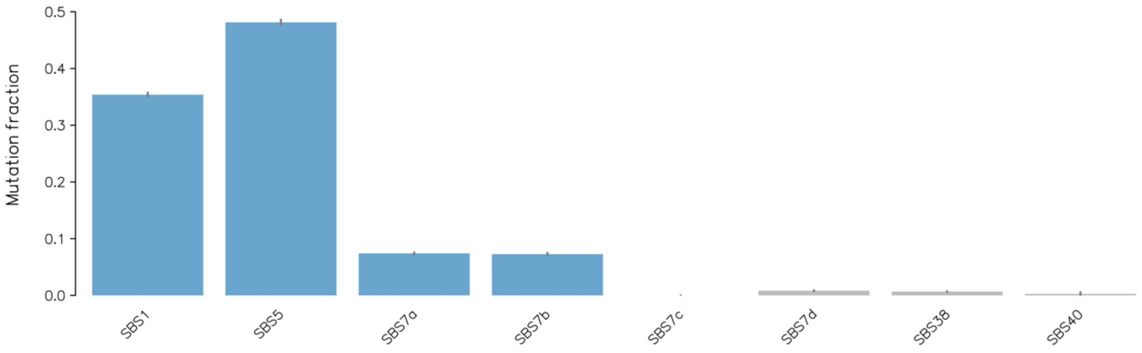

Signature exposures in MM057

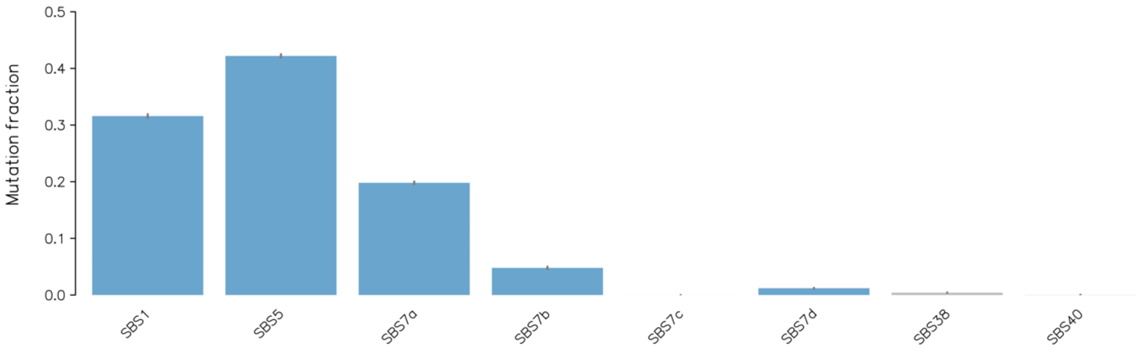

Signature exposures in MM074

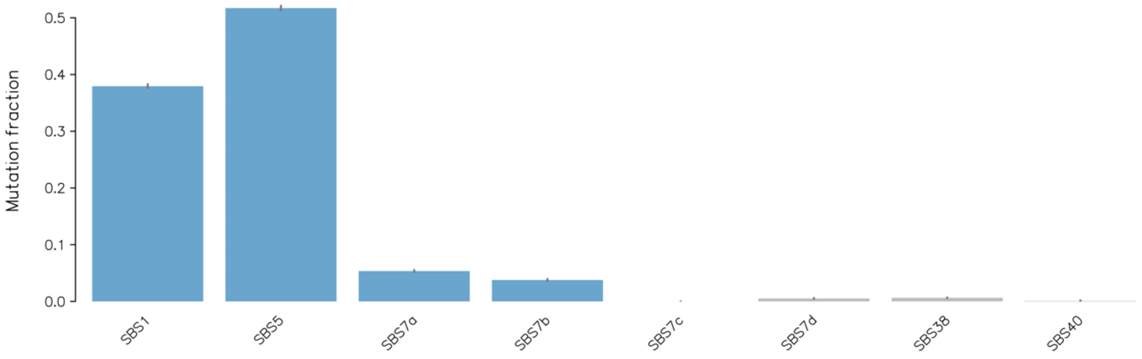

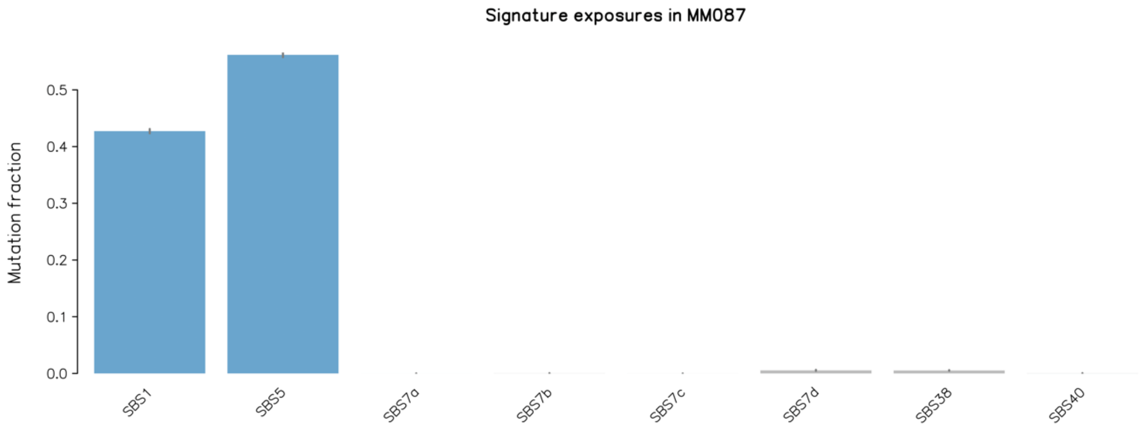
Signature exposures in MM087
Mutation fraction
0.0
0.1
0.2
0.3
0.4
0.5
SBS1
SBS5
SBS7a
SBS7b
SBS7c
SBS7d
SBS38
SBS40

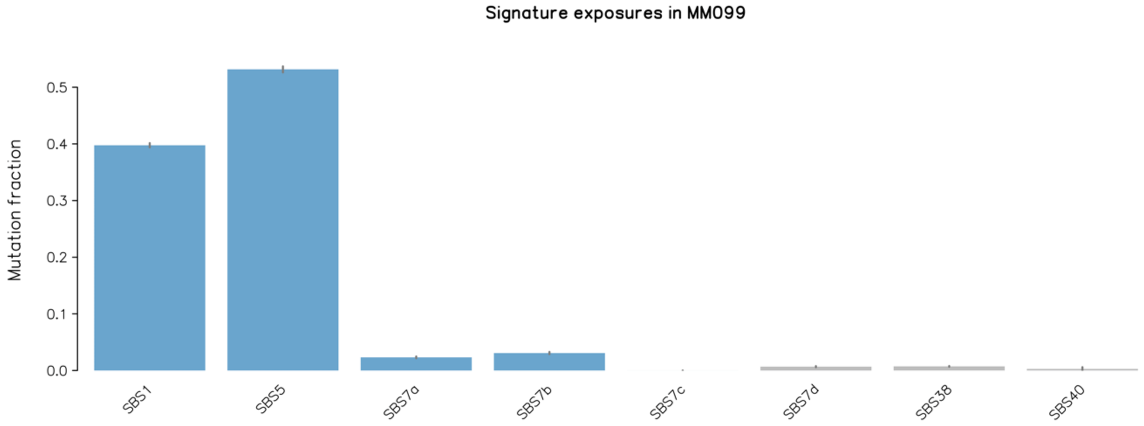
Signature exposures in MM099
Mutation fraction
0.0
0.1
0.2
0.3
0.4
0.5
SBS1
SBS5
SBS7a
SBS7b
SBS7c
SBS7d
SBS38
SBS40

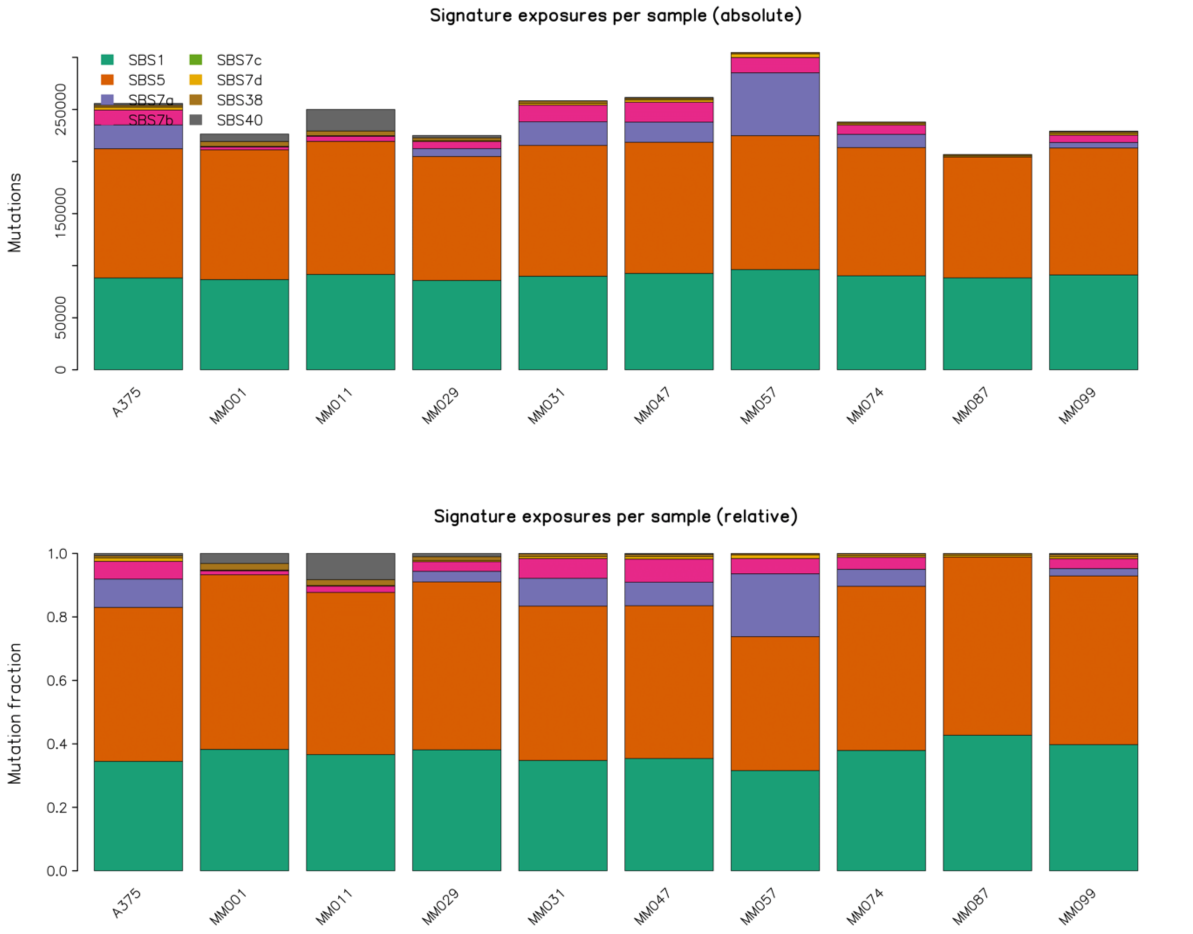

Supplemental Figure S2. Mutation signature analysis of melanoma cell lines. The fraction of somatic SNVs attributable to each mutational signature is indicated.

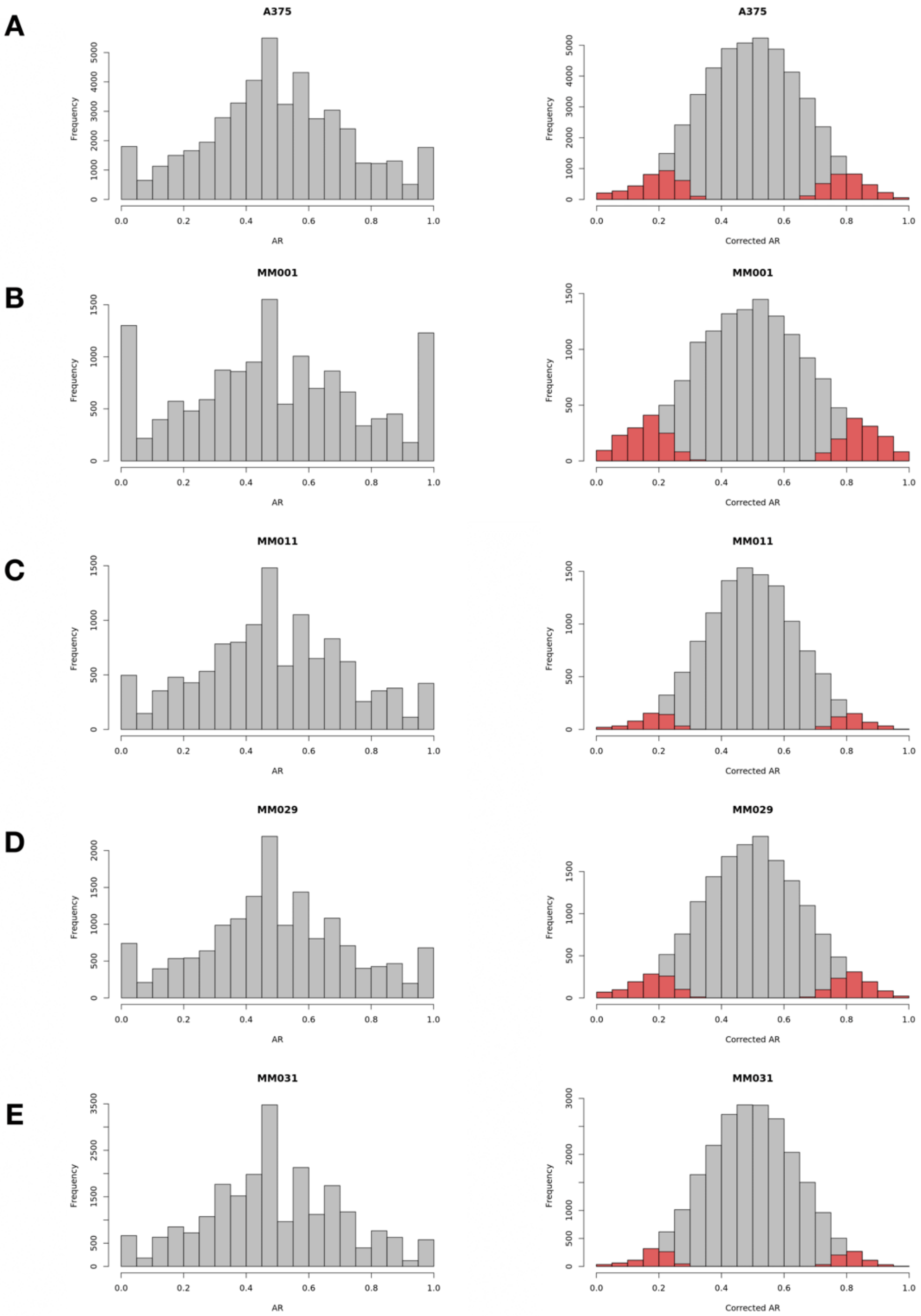
A
A375
Frequency
AR
Corrected AR
B
MM001
C
MM011
D
MM029
E
MM031

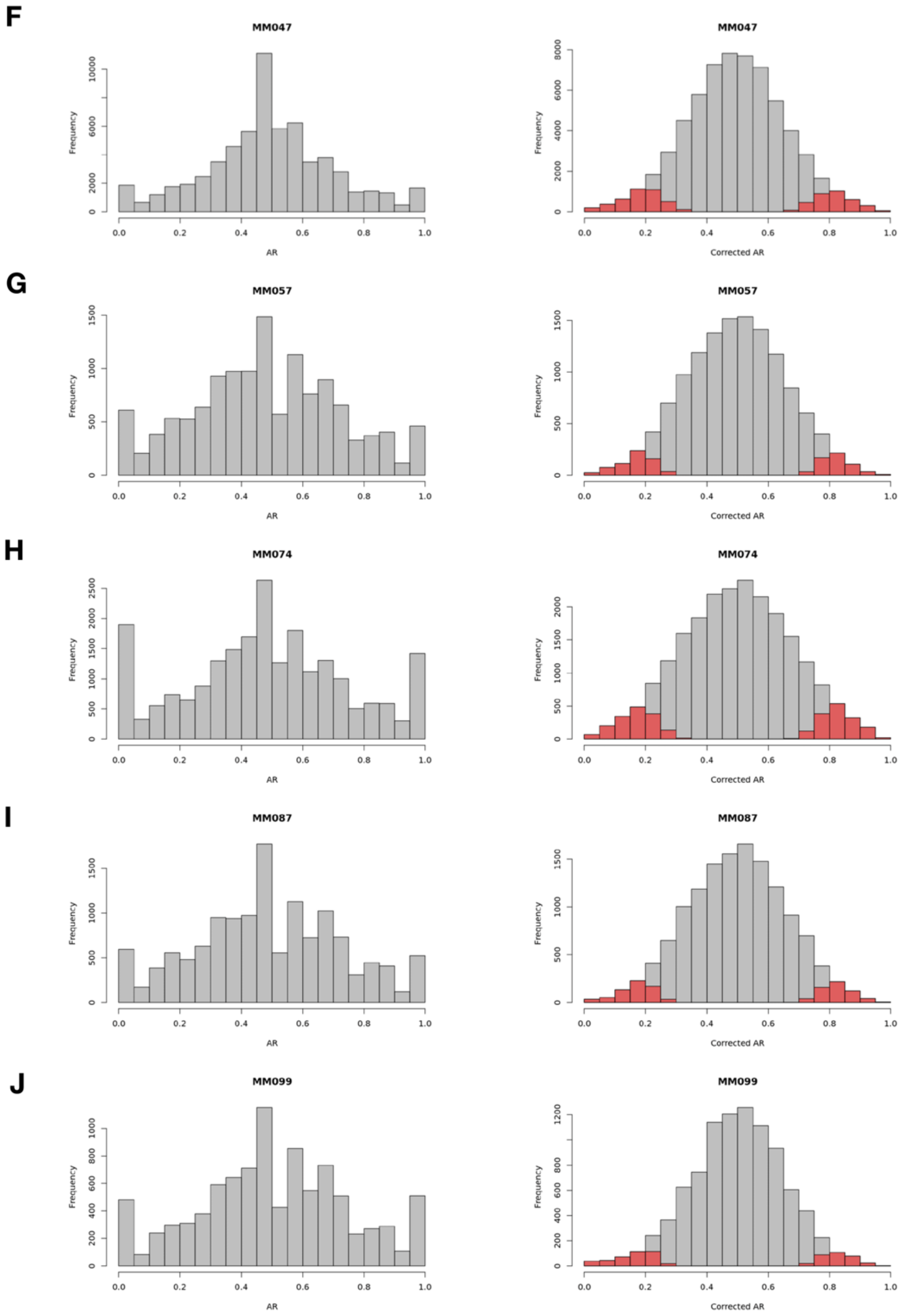

Supplemental Figure S3. Histograms showing the distribution of allelic ratios (AR) before (on the left) and after (on the right) allelic ratio correction using BaalChIP. Corrected allelic ratios are estimated with BaalChIP using genomic allelic ratios from whole genome sequencing data.

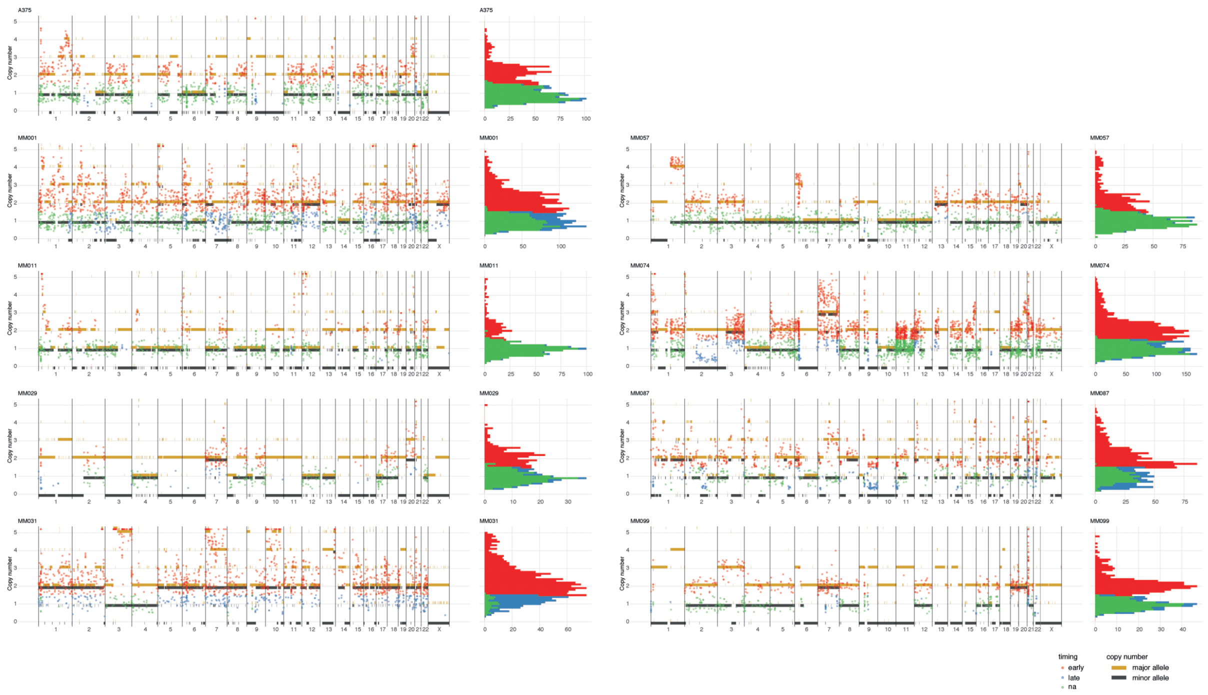

Supplemental Figure S4. Allele-specific and mutation copy number analysis of melanoma cell lines. For each sample, the genome-wide allele-specific copy number is shown. Superposed are the identified ASCAVs in that cell line, of which the mutation copy number is plotted. The colour of the ASCAVs indicates whether they can be classified as either early or late. If their copy number context does not allow timing, they are labelled “na”. Next to each copy number plot is a histogram of the mutation copy number of the identified ASCAVs.

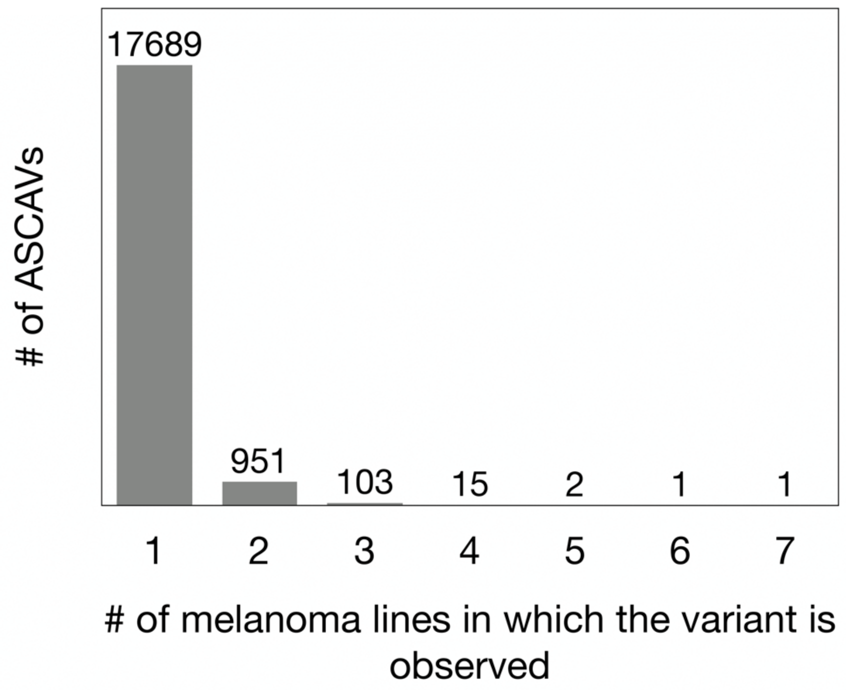

Supplemental Figure S5. Number of shared ASCAVs across ten melanoma lines. Numbers above the bars indicate the number of variants shared between 2 to 7 melanoma lines. 1,073 variants were detected in at least two MM-lines.

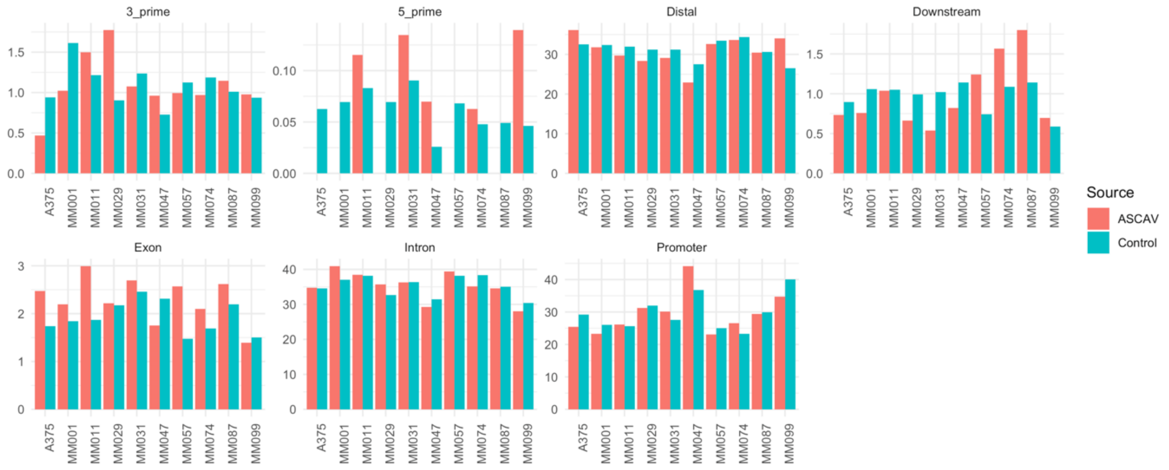

Supplemental Figure S6. Genomic localization of ASCA and control variants. Percentage of variants overlapping various genomic regions are presented in the barplot. ASCA and control variants do not display large differences in genomic localization.

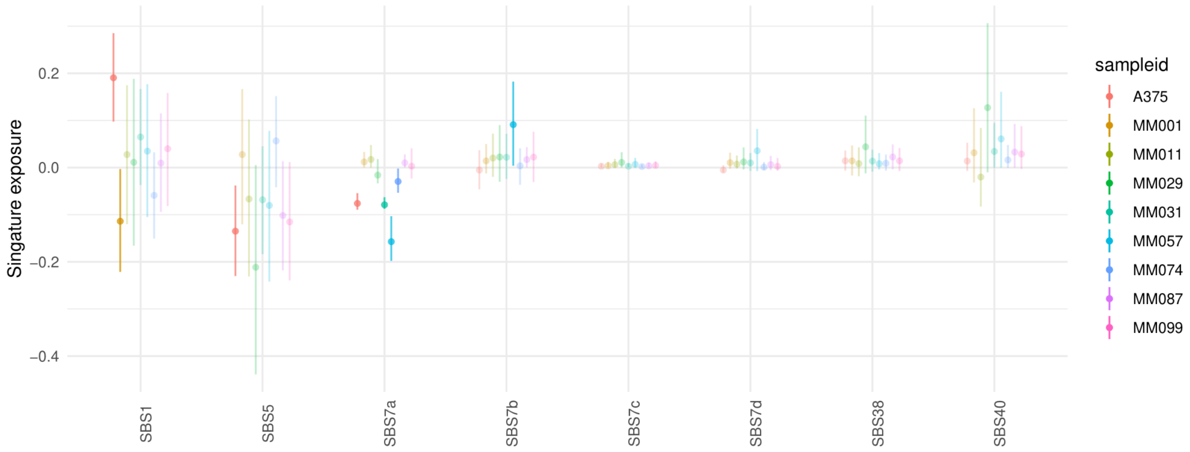

Supplemental Figure S7. Differential mutational signature exposure as estimated from all somatic SNVs minus the exposure estimated from somatic ASCAVs alone. The 95% highest density interval is indicated and samples are highlighted only when the signature has a lower bound contribution estimate > 0.01 and the difference between the two exposures does not overlap 0.

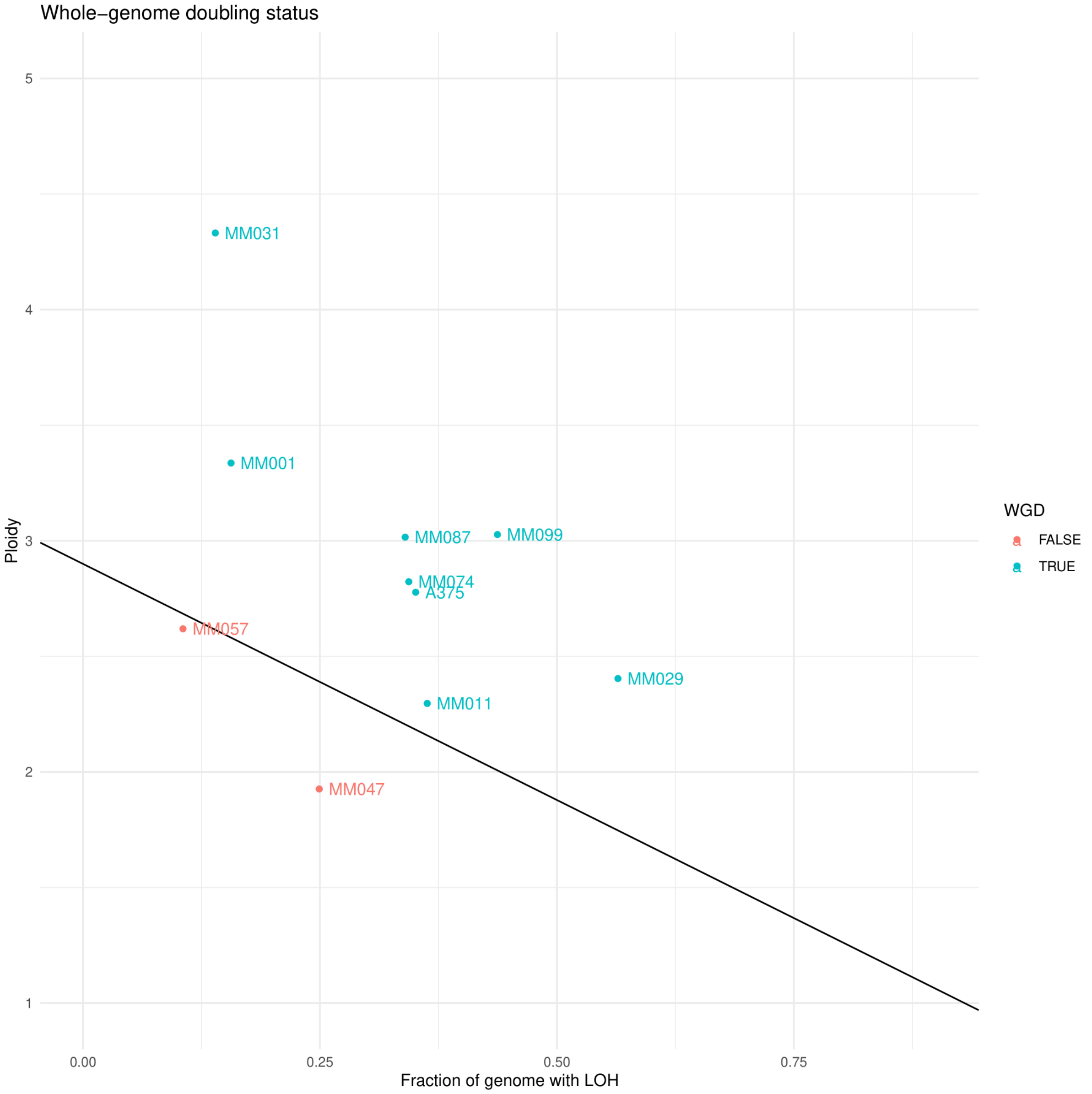

Supplemental Figure S8. Assignment of whole-genome duplication status. Samples are classified as whole-genome doubled using the approach presented in (Dentro et al. 2020). Cases with a high ploidy and a corresponding high degree of loss of heterozygosity are likely to have undergone a whole-genome doubling event.

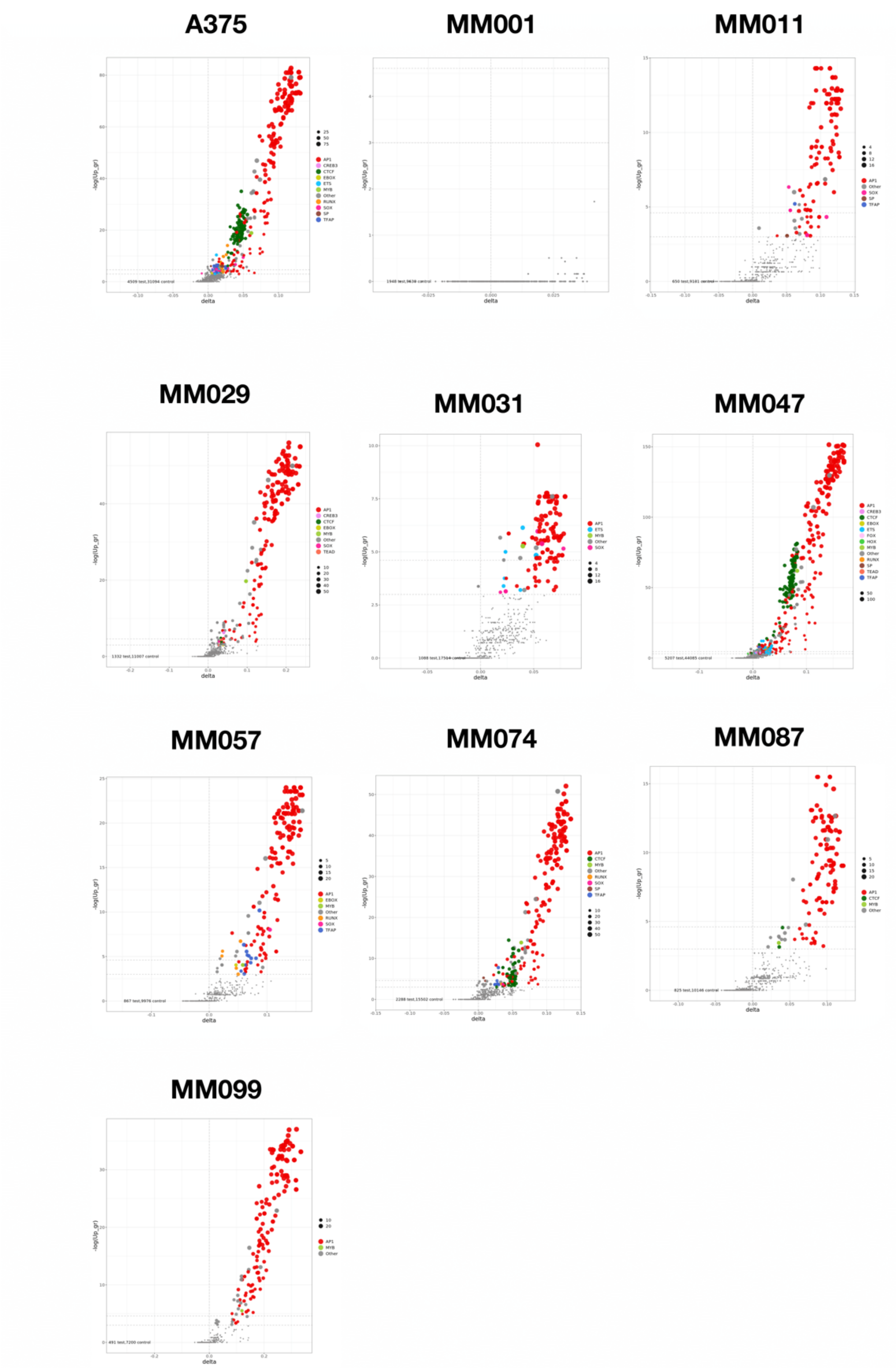

Supplemental Figure S9. Scatter plot of motifs that are associated (positively) with chromatin accessibility. Here the x-axis represents delta cluster-buster motif score, and the y-axis represents the -log10 scaled FDR corrected p-value (calculated by comparing ASCAVs with control SNPs using Fisher's Exact Test). The sizes of the dots are proportional to the number of occurrences of the motif. Each motif is colored based on its direct or inferred similarity to a transcription factor family. MM001 resulted in no significant motif enrichment using this analysis, and it is the only MM line without AP-1 activity, thus changes in AP-1 binding sites due to genomic variation have no effect on chromatin accessibility in MM001.

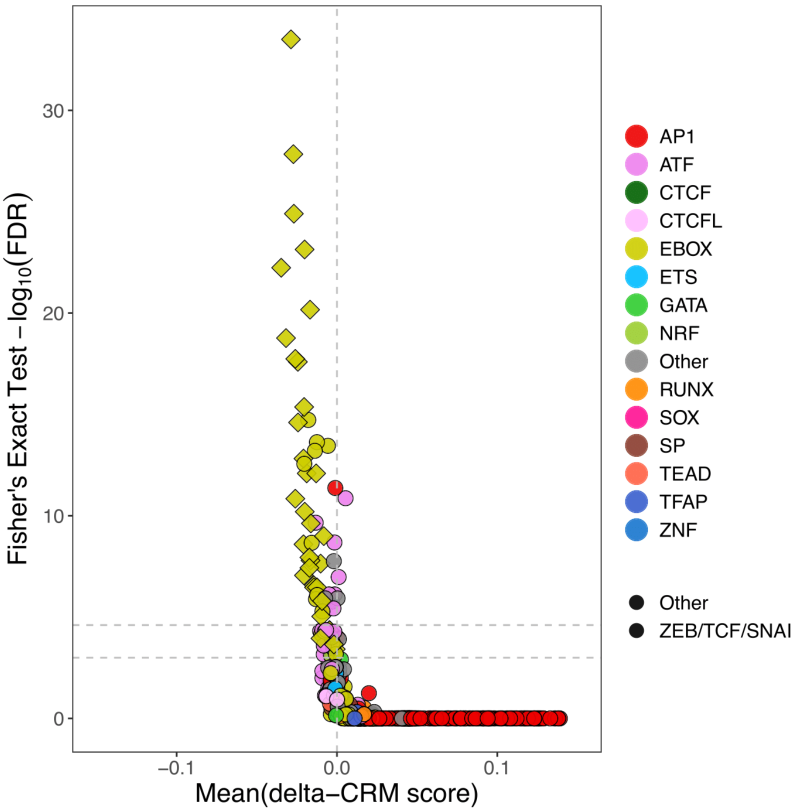

Supplemental Figure S10. Scatter plot of motifs that are associated (negatively) with chromatin accessibility. We tested whether motif gains or losses can be *discordant* with the allelic accessibility imbalance. In other words, a SNP that creates a TF binding site would cause a decrease in accessibility. Here, individual analysis per MM line resulted in no significant motif enrichment, however, due to the increased power in a global analysis across all samples, when all samples are merged, motifs from the ZEB/TCF/SNAI family, which are known repressor transcription factors in the neural crest lineage cells including melanomas (indicated in diamond shapes in the figure). These events are relatively rare and are only observed at 651 allelic imbalanced ATAC-seq peaks.

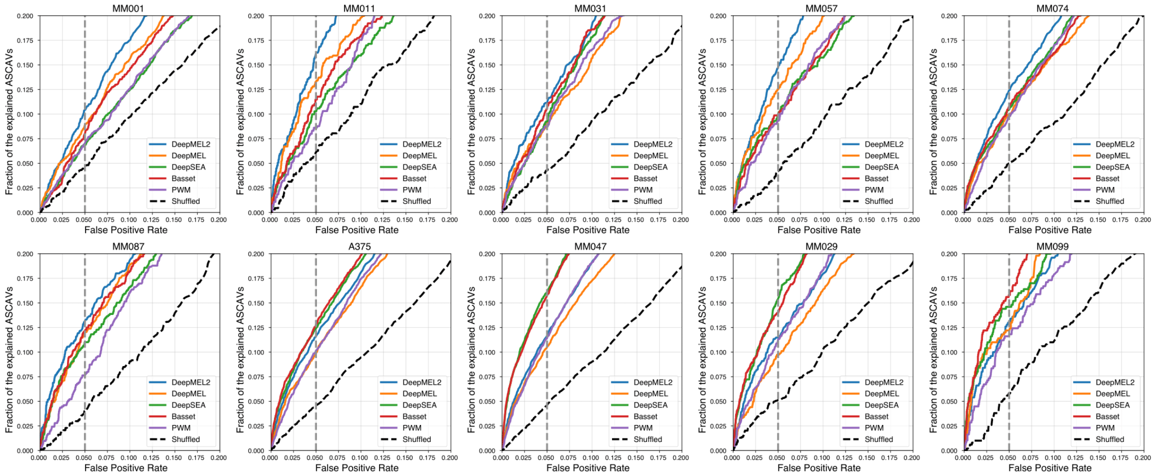

Supplemental Figure S11. Fractions of the explained ASCAVs calculated by using all models at different false positive rates for each MM line are shown as curves. Dashed black line represent control for each MM line, where the labels of the ASCAVs and the control variants are shuffled.

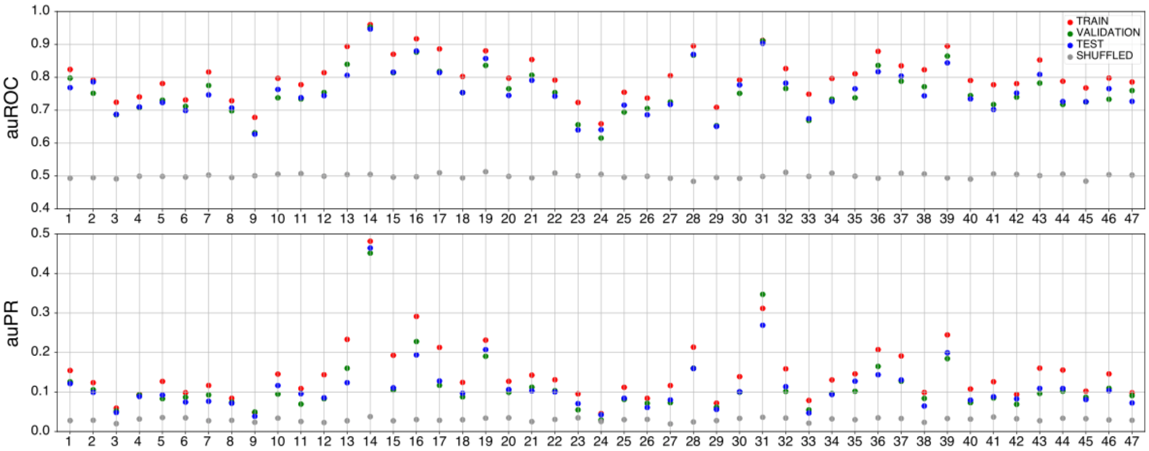

Supplemental Figure S12. Area under the ROC (top) and the PR (bottom) curve values of DeepMEL2 on 47 topics are shown for training (%80), validation (%10), and test (%10) sets. auROC and auPR values calculated on training data when its labels are shuffled are shown as grey.

A

Feature	Logo	NES	Possible TFs
**JUN**			
hocomoco__JUN_HUMAN.H10MO.A		14.63247	AP1
cisbp__M6319		14.55501	AP1
homer__DATGASTCAT_BATF		14.32288	AP1
homer__ATGACTCATC_AP-1		14.09689	AP1
cisbp__M6317		14.00784	AP1
**FOSL1**			
cisbp__M6319		12.53051	AP1
hocomoco__JUN_MOUSE.H11MO.0.A		12.51691	AP1
hocomoco__FOS_HUMAN.H11MO.0.A		12.45470	AP1
hocomoco__JUNB_MOUSE.H11MO.0.A		12.37291	AP1
hocomoco__FOSB_MOUSE.H11MO.0.A		12.34499	AP1
**FOS**			
hocomoco__JUN_MOUSE.H11MO.0.A		12.74043	AP1
hocomoco__FOS_HUMAN.H11MO.0.A		12.73271	AP1
cisbp__M6319		12.73174	AP1
hocomoco__JUNB_MOUSE.H11MO.0.A		12.69791	AP1
hocomoco__FOSB_MOUSE.H11MO.0.A		12.64592	AP1
**JUNB**			
hocomoco__JUNB_MOUSE.H11MO.0.A		13.19177	AP1
transfac_pro__M08936		13.05580	AP1
transfac_pro__M08926		13.00121	AP1
cisbp__M6319		12.85766	AP1
hocomoco__FOSB_MOUSE.H11MO.0.A		12.80070	AP1

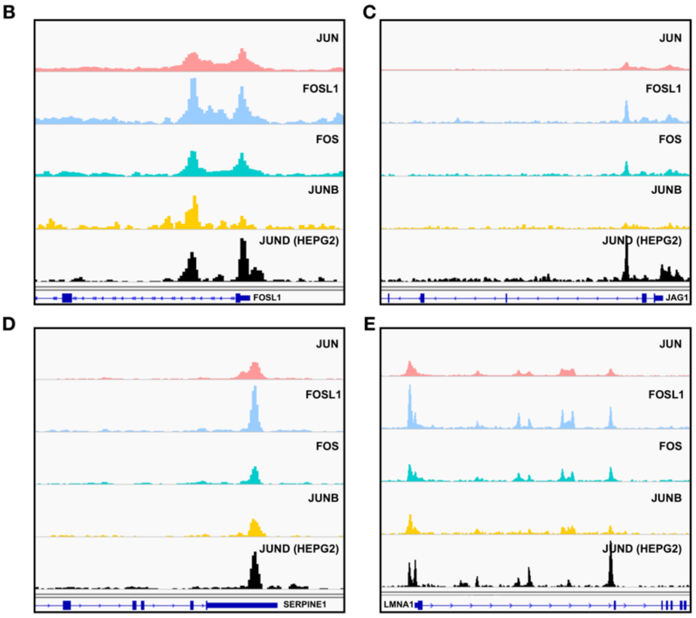

Supplemental Figure S13. A. The table shows the top 5 motifs enriched in each ChIP-seq experiment using i- cisTarget. **B-E.** shows IGV screenshots of the ChIP-seq signal of each AP-1 factor profiled in MM099 together with JUND ChIP-seq signal from HEPG2 cell line (ENCSR000EEI).

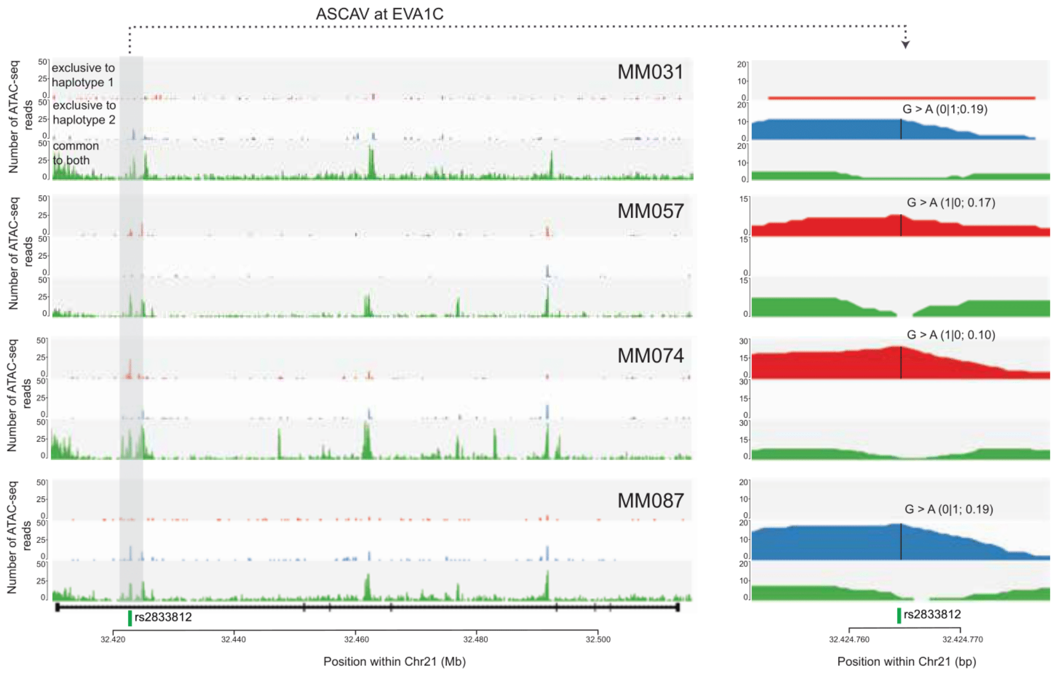

Supplemental Figure S14. Haplotype resolved visualization of EVA1C across four melanoma lines with harboring rs2833812. The SNP in the first intron of EVA1C results in allelic imbalance in all lines where the SNP is present. For each MM line, the genotype prediction from WGS and corrected reference allele fraction is displayed in the parenthesis.

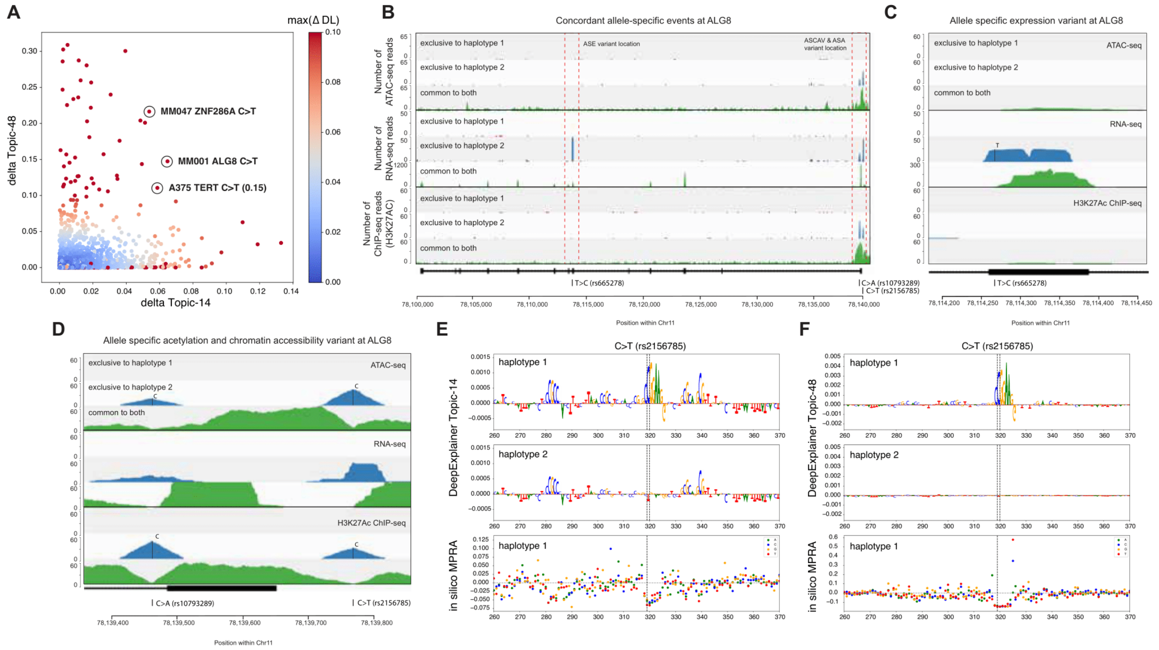

Supplemental Figure S15. A. Scatter plot of delta Topic-14 score (promoter topic) vs. delta Topic-48 score (GABPA topic) of all ASCAVs from 10MM lines calculated by using the DeepMEL2+GABPA model. ASCAVs are colored by their maximum delta prediction score. The ALG8 C>T mutation that MM001 has as a significant ASCAV is shown as a black circle on the scatter plot. **B.** Concordant allele-specific events at ALG8 gene are shown with haplotype resolved ATAC-seq, RNA-seq and H3K27ac ChIP-seq reads. For each data type, reads mapping to haplotype 1 is shown with red, haplotype 2 with blue and commonly mapping reads with green. The box on the left hand side (indicated with a dashed red line) is expanded in panel c and contains an allele-specific expression (ASE) variant. The box on the right hand side is expanded in panel d and contains variants resulting allele-specific chromatin accessibility and acetylation. **C.** Haplotype resolved visualization of the ASE variant (rs665278) and ALG8 gene. **D.** Haplotype resolved visualization of the ASCAV and ASHV at ALG8 gene. **E, F.** DeepExplainer plots of MM001 ALG8 C>T variant by using Topic-14 (**E**) and Topic-48 (**F**) are shown for each haplotype. C>T variant destroys the ETS transcription factors binding site on haplotype 1 of MM001.

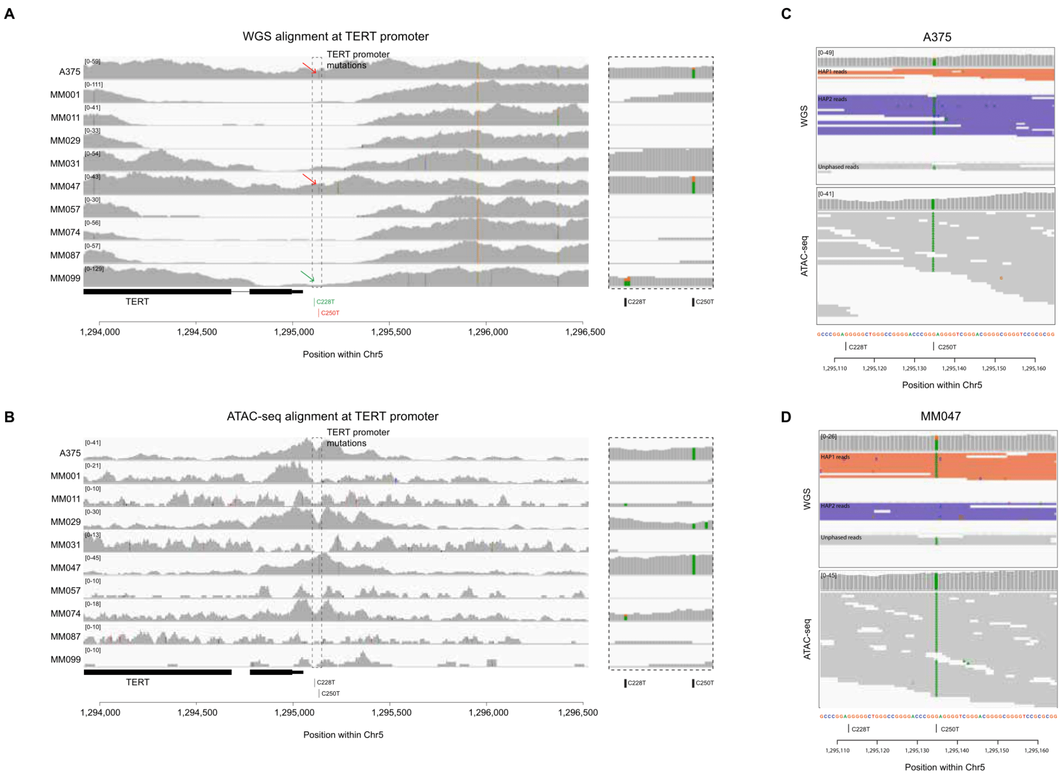

Supplemental Figure S16. A. IGV screenshot showing WGS data of 10 melanoma lines at the TERT promoter region. Arrows indicate the samples with C228T (green) and C250T (red) mutations, respectively. Inset shows the loci with the mutations. A375 and MM047 samples harbor heterozygous C250T mutation, while MM099 harbors a heterozygous C228T mutation. **B.** IGV screenshot showing ATAC- seq data of 10 melanoma lines at the TERT promoter region. MM099 had no detectable peak at the TERT promoter, while MM047 and A375 ATAC-seq reads harbored the C250T mutation homozygously. Inset shows the loci with the mutations. **C.** IGV screenshot showing WGS and ATAC-seq reads of A375 at C250T locus. Here, the WGS reads colored based on their phasing (haplotype 1: orange, haplotype 2: purple, unphased: grey). **D.** IGV screenshot showing WGS and ATAC-seq reads of MM047 at C250T locus.

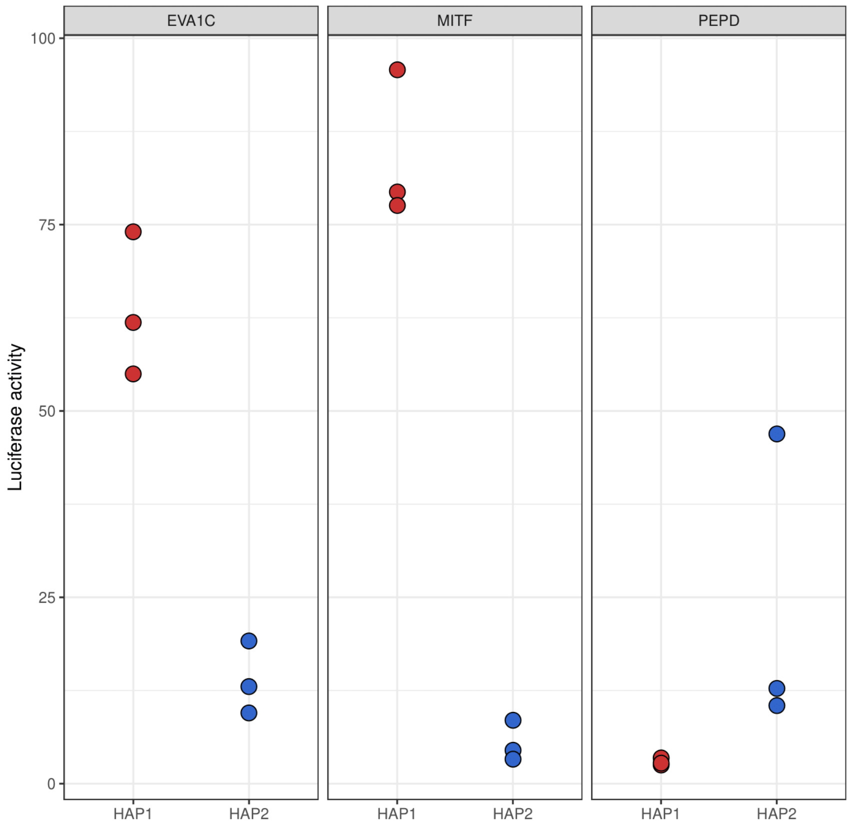

Supplemental Figure S17. Individual luciferase activity values for all three replicates of the tested ASCAVs in MM057.
