# Supplementary material for: Interpretation of allele-specific chromatin accessibility using cell state–aware deep learning
Source: Genome Res. 2021 Jun;31(6):1082–96. doi: 10.1101/gr.260851.120 (PMC8168584; doi:10.1101/gr.260851.120)
Supplement: Supplemental Material [file supp_gr.260851.120_Supplemental_Methods.pdf]

Supplemental Methods

Cell culture

Cells were cultured in Ham's F10 nutrient mix (Thermo Fisher Scientific) supplemented with 10% fetal bovine serum (Invitrogen) and 100 µg/ml penicillin/streptomycin (Thermo Fisher Scientific). The commonly-used, human melanoma cell line A375 was obtained from the ATCC and was maintained in Dulbecco's Modified Eagle's Medium with high glucose and GlutaMAX (Thermo Fisher Scientific), supplemented with 10% fetal bovine serum and penicillin-streptomycin. Cell cultures were kept at 37°C, with 5% CO_2 and were regularly tested for mycoplasma contamination, and were found negative. For each cell line, 1 million cells were used as input for the extraction of genomic DNA.

Phased whole genome library preparation and sequencing

Briefly, first, HMW gDNA was isolated using the MagAttract HMW DNA Kit (Qiagen). Quality of the HMW gDNA (length and integrity) was verified using the Genomic DNA ScreenTape on the Tapestation instrument (Agilent). Next, the HMW gDNA was carefully, serially diluted to final concentrations of 0.8 – 1.0 ng/µl (measured using the Qubit High Sensitivity kit), before loading onto the microfluidic Genome Chip (10x Genomics). After generation of nanoliter-scale Gel bead-in-EMulsions (GEMs), genomic fragments were barcoded during isothermal incubation at 30°C in a C1000 Touch Thermal Cycler (Bio Rad). After incubation, the droplets were broken and the pooled barcoded fragments cleaned with a Cleanup Mix containing DynaBeads (Thermo Fisher Scientific). Next, the fragments were end-repaired, A-tailed and index adaptor ligated, with cleanup in between steps using SPRIselect Reagent Kit (Beckman Coulter). The Post-ligation product was then amplified with a C1000 Touch Thermal Cycler during the Sample Index PCR. Finally, the sequencing-ready library was cleaned up with SPRIselect beads. Libraries were sequenced on a NovaSeq6000 S2 flow cell with paired-end 2 x 150 cycli.

Sequence reads were processed using 10x Genomics companion software longranger (v2.2.2). Short variant calling was performed with GATK (v3.5) using --vcmode gatk option in longranger. Single nucleotide variants and indels were further filtered with a depth of sequencing filter of 20.

Copy number analysis

Briefly, allele counts (base quality ≥ 20 and mapping quality ≥ 35) were obtained at the 1000 Genomes project hg38 SNP loci using alleleCount v4.1.0 (https://github.com/cancerit/alleleCount) and transformed into B-allele frequencies (BAF) and LogR. Loci meeting a minimal read depth of 10 were passed along to ASCAT, which was run in tumor-only mode with correction for GC-content.

Whole-genome doubling status of the cell lines was assigned based on their ploidy (average genome-wide copy number) and fraction of the genome exhibiting loss of

heterozygosity (LOH), as described by (Dentro et al. 2020). Cases with a high ploidy and corresponding high degree of LOH are likely to have undergone a whole-genome doubling event.

Personalized genome construction

Briefly, we partitioned the reference genome into 3kb pieces per chromosome and mapped to personalized genomes with BLAT using the following options: -tileSize=11 -fastMap -minIdentity=95 -noHead -minScore=100. Resulting alignment files in psl format were combined, and chained with axtChain command and then sorted with chainMergeSort command. Then, alignment nets were created from chain files using chainNet command, and finally liftOver chain files were created with netChainSubset command.

ATAC-seq library preparation and sequencing

Cells were washed, trypsinized, spun down at 1000 RPM for 5 min, medium was removed and the cells were resuspended in 1 mL medium. Cells were counted and experiments were only continued when a viability of above 90% was observed. 50,000 cells were pelleted at 500 RCF at 4°C for 5 min, medium was carefully aspirated and the cells were washed and lysed using 50 uL of cold ATAC-Resupension Buffer (RSB) (see Corces et al., 2017 for composition) containing 0.1% NP40, 0.1% Tween-20 and 0.01% digitonin by pipetting up and down three times and incubating the cells on ice for 3 min. 1 mL of cold ATAC-RSB containing 0.1% Tween-20 was added and the eppendorf was inverted three times. Nuclei were pelleted at 500 RCF for 10 min at 4°C, the supernatant was carefully removed and nuclei were resuspended in 50 uL of transposition mixture (25 uL 2x TD buffer (see Corces et al., 2017 for composition), 2.5 uL transposase (100 nM), 16.5 uL DPBS, 0.5 uL 1% digitonin, 0.5 uL 10% Tween-20, 5 uL H2O) by pipetting six times up and down, followed by 30 minutes incubation at 37°C at 1000 RPM mixing rate. After MinElute clean-up and elution in 21 uL elution buffer, the transposed fragments were pre-amplified with Nextera primers by mixing 20 uL of transposed sample, 2.5 uL of both forward and reverse primers (25 uM) and 25 uL of 2x NEBNext Master Mix (program: 72°C for 5 min, 98°C for 30 sec and 5 cycles of [98°C for 10 sec, 63 °C for 30 sec, 72°C for 1 min] and hold at 4°C). To determine the required number of additional PCR cycles, a qPCR was performed (see Buenrostro et al., 2015 for the determination of the number of extra cycles). The final amplification was done with the additional number of cycles, samples were cleaned-up by MinElute and libraries were prepared using the KAPA Library Quantification Kit as previously described (Corces et al. 2017). Samples were sequenced on a HiSeq 4000 or NextSeq 500 High Output chip.

ATAC-seq peak calling

Briefly, peak scores within each peak-set were standardized by dividing peak scores to the sum of all peak scores for that sample. Haplotype 1 and 2 mapped peak sets lifted over the reference genome and consolidated peak set per sample obtained by an iterative filtering strategy: peaks were ranked by their normalized peak score, and any peak that overlapped with the highest scoring peak was filtered out. Next, the second highest scoring peak is processed this way, and the same procedure is repeated until a non-overlapping peak set is obtained. This strategy enabled us to obtain 500bp fixed-width peaks that are not biased towards the reference genome (referred to as consolidated peak sets).

Luciferase assays

Briefly, 125,000 cells were seeded per well of a 24 well plate. The following day, cells were transfected with 40 ng *Renilla* plasmid + 400 ng pGL4.23 plasmid using Lipofectamine 2000 (Invitrogen). 48 hours following transfection, cell lysates were prepared with Passive Lysis Buffer and Luciferase activity was measured on a VICTOR X3 plate reader (PerkinElmer). Luciferase assay values are represented in fold change of Fluc (pGL4.23 plasmid) vs Rluc (*Renilla* plasmid). The assay was performed on three biological replicates (**Figure S17**).

AP1 ChIP-seq library preparation and sequencing

Per sample 5-20 ng of precipitated DNA was used to perform library preparation according to the Illumina TruSeq DNA sample preparation guide. In brief, the immunoprecipitated DNA was end-repaired, A-tailed and ligated to diluted sequencing adapters (1/100). After PCR amplification (15 cycles) and bead purification (Agencourt AmpureXP, Analis), the libraries with fragment size of 200-600 bp were sequenced using NovaSeq 6000 (Illumina).
